# Two Kelvin Operation of Ultrawide-Bandgap β‑Ga_2_O_3_ FinFETs and Logic Inverter Integrated Circuits

**DOI:** 10.1021/acs.nanolett.5c06155

**Published:** 2026-02-25

**Authors:** Vishal Khandelwal, Glen Isaac Maciel García, Mritunjay Kumar, Francesco Blanda, Na Xiao, Dongxing Zheng, Ganesh Mainali, Manoj Kumar Rajbhar, Xiao Tang, Xixiang Zhang, Xiaohang Li

**Affiliations:** † Advanced Semiconductor Laboratory (ASL), Electrical and Computer Engineering Program, Computer, Electrical, and Mathematical Sciences and Engineering (CEMSE), 127355 King Abdullah University of Science and Technology (KAUST), Thuwal 23955-6900, Kingdom of Saudi Arabia; ‡ Experimental Spintronics & Low Dimensional Materials and Physics Laboratory, Material Science and Engineering, Physical Science and Engineering Division (PSE), King Abdullah University of Science and Technology (KAUST), Thuwal 23955-6900, Kingdom of Saudi Arabia

**Keywords:** Cryogenic electronics, Ultrawide bandgap, Ga_2_O_3_, Inverter, Integrated circuit, FinFET, Impurity band conduction

## Abstract

Resilient extreme-temperature electronics are critical for applications ranging from quantum computing to space exploration. Ultrawide-bandgap (UWBG) β-Ga_2_O_3_ semiconductors are promising for operation across cryogenic and high-temperature regimes; however, their cryogenic performance remains insufficiently explored. Here, we demonstrate β-Ga_2_O_3_ transistor operation down to 2 K by exploiting Mott’s variable-range hopping (VRH) conduction in impurity bands. The β-Ga_2_O_3_ FinFETs exhibit enhancement-mode behavior with a threshold voltage of 1.87 V, an ON/OFF current ratio exceeding 10^6^, and a subthreshold swing of 152 mV/dec at 2 K. Furthermore, a monolithic β-Ga_2_O_3_ inverter integrated circuit is realized, achieving a voltage swing of 4.88 V and a voltage gain of 28 at a 5 V supply with DC power dissipation of 0.13 μW at 2 K. Stable cryogenic performance arises from FinFET architecture and precise doping that enable VRH, consistent with a two-band transport model of the β-Ga_2_O_3_ channel, hence establishing β-Ga_2_O_3_ cryogenic electronics.

## Graphical abstract

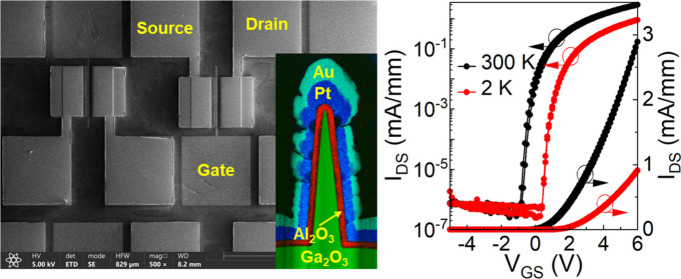

Electronic devices capable of operating in extreme temperatures are highly desirable for a wide application range including cryogenic electronics,^1^ automotive engine control,^2^ downhole drilling,^3^ and space exploration.^4^ For instance, quantum computers require cryogenic electronics, working at a low temperature of 4 K.^5^ Electronics involved in space systems are exposed to large variations in temperature, ranging from 90 to 700 K on Mercury’s surface and 100 to 400 K on the Moon’s surface.^6–8^ Typically, electronic circuits operating in these environments are maintained under thermal control or shielding,^9^ which leads to large system size and weight with increased costs. A viable solution involves realizing electronics that are resilient to both extremes: cryogenic and high temperatures. Implementing electronic systems with components and circuits inherently resistant to extreme temperatures can reduce the burden of deploying separate temperature-specific units and protection, enabling a compact and cost-efficient solution.^10^

Developing extreme-temperature electronics using wide-bandgap (WBG) and ultrawide-bandgap (UWBG) semiconductors could be advantageous compared to silicon, as they already exhibit a stable device performance at high temperatures (HT) up to 500 °C owing to low intrinsic carrier concentrations.^11–13^ However, the cryogenic operation of WBG semiconductors shows degraded device performance due to carrier freeze-out. For instance, WBG silicon carbide (SiC) and bulk gallium nitride (GaN) exhibit carrier freeze-out near 100 K.^14,15^ This causes a substantial increase in on-resistance (*R*_*ON*_) and a higher threshold voltage (*V*_*TH*_) in transistor characteristics. The polarization-induced two-dimensional carrier gas in the GaN-based heterostructure is free of carrier freeze-out.^8^ However, it is limited to only lateral device structures and also exhibits the kink effect near 100 K.^16^

Nonetheless, UWBG beta-gallium oxide (β-Ga_2_O_3_) semiconductors hold promise for cryogenic temperatures (CTs) due to their demonstration of the no electron freeze-out properties down to 2 K in Sn-doped β-Ga_2_O_3_ native substrates.^17^ Another study on β-Ga_2_O_3_ epitaxial films shows no electron freeze-out down to 40 K.^18^ This observation indicates the potential of β-Ga_2_O_3_ devices for cryogenic operation, presenting significant additional advantages. (1) Because of its bulk cryogenic property, both lateral and vertical β-Ga_2_O_3_ devices such as diodes and transistors can be realized down to 2 K.^19,20^ (2) High-quality and scalable β-Ga_2_O_3_ epitaxial films on low-cost melt-grown native substrates suggest its applicability for various devices, including transistors, diodes, logic circuits, and drivers. This can further facilitate the development of a monolithic integrated highly compact cryogenic chip with vast on-chip functionalities.^20,21^ (3) Ga_2_O_3_, with a UWBG of ∼4.8 eV and a large critical electric field (*E*_*critical*_) of ∼8 MV/cm, holds significance for cryogenic power electronics.^22^ (4) With the cryogenic operation, β-Ga_2_O_3_ can be an excellent choice for interplanetary space probes, where its exceptional high-temperature (HT) device operation^13,23^ with radiation hardness^24^ is a crucial factor. To fully harness these advantages and develop extreme temperature electronics using β-Ga_2_O_3_, the realization of cryogenic operation of β-Ga_2_O_3_ devices is essential, which has not been reported so far.

In this work, we report on the enhancement (E-) mode β-Ga_2_O_3_ fin field effect transistors (FinFETs) with a *V*_*TH*_ of 1.87 V, a high current on–off ratio (*I*_*ON*_ */I*_*OFF*_) > 10^6^, and a subthreshold swing (*SS*) of 152 mV/dec at 2 K. Small temperature variations were observed in key device characteristics, such as the *SS*, which changed by only 11 mV/dec, and a 3.3 times decrease in *I*_*ON*_ */I*_*OFF*_ at 2 K compared to 300 K. Subsequently, monolithically integrated n-channel metal oxide semiconductor (NMOS) inverter ICs were demonstrated at 2 K, showing a logic voltage swing of 4.88 V and a voltage gain of 28. This work presents four notable achievements, which are not reported in previous literature: (1) first UWBG transistors and logic inverters operation at CTs; (2) first cryogenic operation of UWBG FinFETs; (3) material and device engineering for stable electrical characteristics across temperatures; and (4) correlation between FinFET′ electrical characteristics and material properties.

Figure 1(a) and (b) show the 3D schematic and top scanning electron microscope (SEM) view of the β-Ga_2_O_3_ FinFETs on semi-insulating (010) β-Ga_2_O_3_ substrates. FinFET architecture is employed to enable stable and high-performance E-mode operation at CTs, facilitating further on-chip functionality and miniaturized circuit design. Additional discussion can be found in the Supporting Information (Section I). The FinFETs were fabricated on a homoepitaxial 600 nm Si-doped β-Ga_2_O_3_ film grown by pulsed laser deposition (PLD). The total number of fins in one FinFET is 55, with a channel width (*W*_*CH*_) of 70 μm (Section II, Supporting Information), channel length (*L*_*CH*_) of 10 μm, and gate length (*L*_*G*_) of 4 μm, as shown in Figure 1(c). Further details on β-Ga_2_O_3_ growth and device fabrication can be found in the Experimental Section (Section III, Supporting Information). The as-grown Si-doped β-Ga_2_O_3_ film shows excellent material quality with a smooth surface morphology of RMS roughness of 1.1 nm, shown in Figure 1(d). The inset shows the rocking curve full-width half-maximum (RC-fwhm) of 105 arcsecs of the β-Ga_2_O_3_ (020) plane.

**Figure 1:**
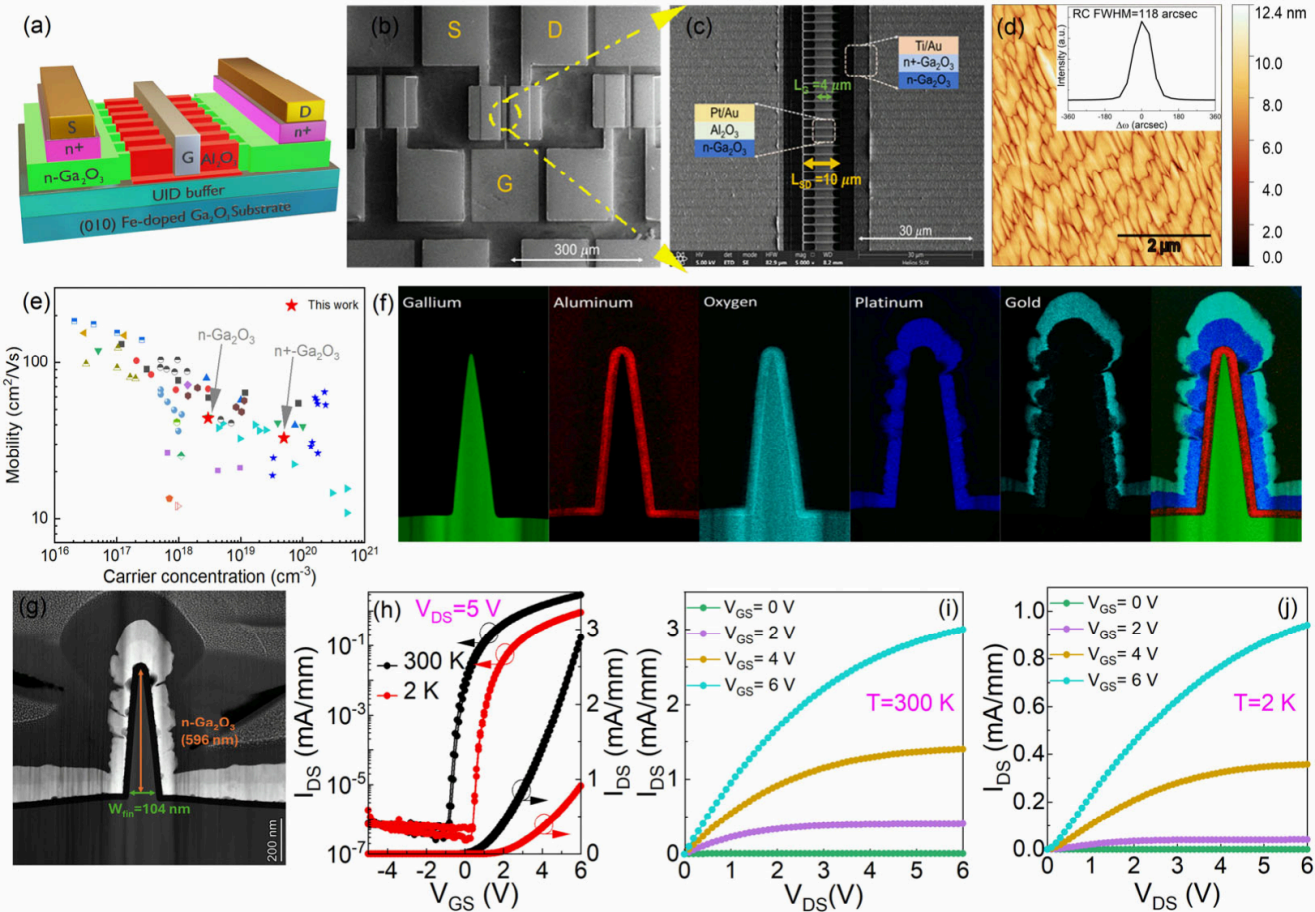
β-Ga_2_O_3_ FinFETs: (a) 3D schematic. (b) Top SEM view, highlighting the source (S), drain (D), and gate (G). (c) Zoom-in image of the fin area, showing *L*_CH_ = *L*_SD_ = 10 μm, *L*_G_ = 4 μm. Si-doped β-Ga_2_O_3_ film: (d) atomic force microscopy (AFM) surface morphology with an RMS roughness of 1.1 nm; the inset shows an X-ray diffraction (XRD) omega scan of the (020) peak, depicting an RC-fwhm of 105 arcsec. (e) Benchmark of the PLD-grown β-Ga_2_O_3_ films for n– Ga_2_O_3_ and n+ Ga_2_O_3_ compared to the literature. (f) EELS images of the elements of interest. (g) TEM image of the cross-section of the fin, depicting constituent layers with fin dimensions. (h) Transfer curve at 300 and 2 K at *V*_*DS*_ = 5 V. Output curves at different *V*_*GS*_ for (i) 300 and (j) 2 K, respectively.

The doping control of β-Ga_2_O_3_ thin films is crucial. In this study, we have developed two doping profiles: *n*– Ga_2_O_3_ for FinFET channels and *n+* Ga_2_O_3_ for contacts, respectively. For the channels, the electron concentration (*n*) and mobility (μ) are 2.3 × 10^18^ cm^–3^ and 37.0 cm^2^/(V s) at 300 K, respectively, whereas, for the ohmic contacts, the *n* and μ are 4.9 × 10^19^ cm^–3^ and 33.3 cm^2^/(V s) at 300 K measured by Hall-effect measurement. Figure 1(e) shows the benchmarking with reported *n*’s and μ’s of n– β-Ga_2_O_3_, showing comparable Si-doping control with state-of-the-art results.^25^ The values of *n* and μ at 2 K are 2.27 × 10^18^ cm^–3^ and 8.8 cm^2^/(V s), discussed in Figure 3(a). Figure 1(f) shows the electron energy loss spectroscopy (EELS) measurement, depicting a conformal and sharp interface of an Al_2_O_3_ dielectric and metal on the triangular Ga_2_O_3_ fin, as observed from the transmission electron microscopy (TEM) image (Figure 1(g)). Each β-Ga_2_O_3_ fin has a fin width (*W*_*fin*_) of 104 nm with a depth of 596 nm, suggesting an aspect ratio of ∼6.

**Figure 2:**
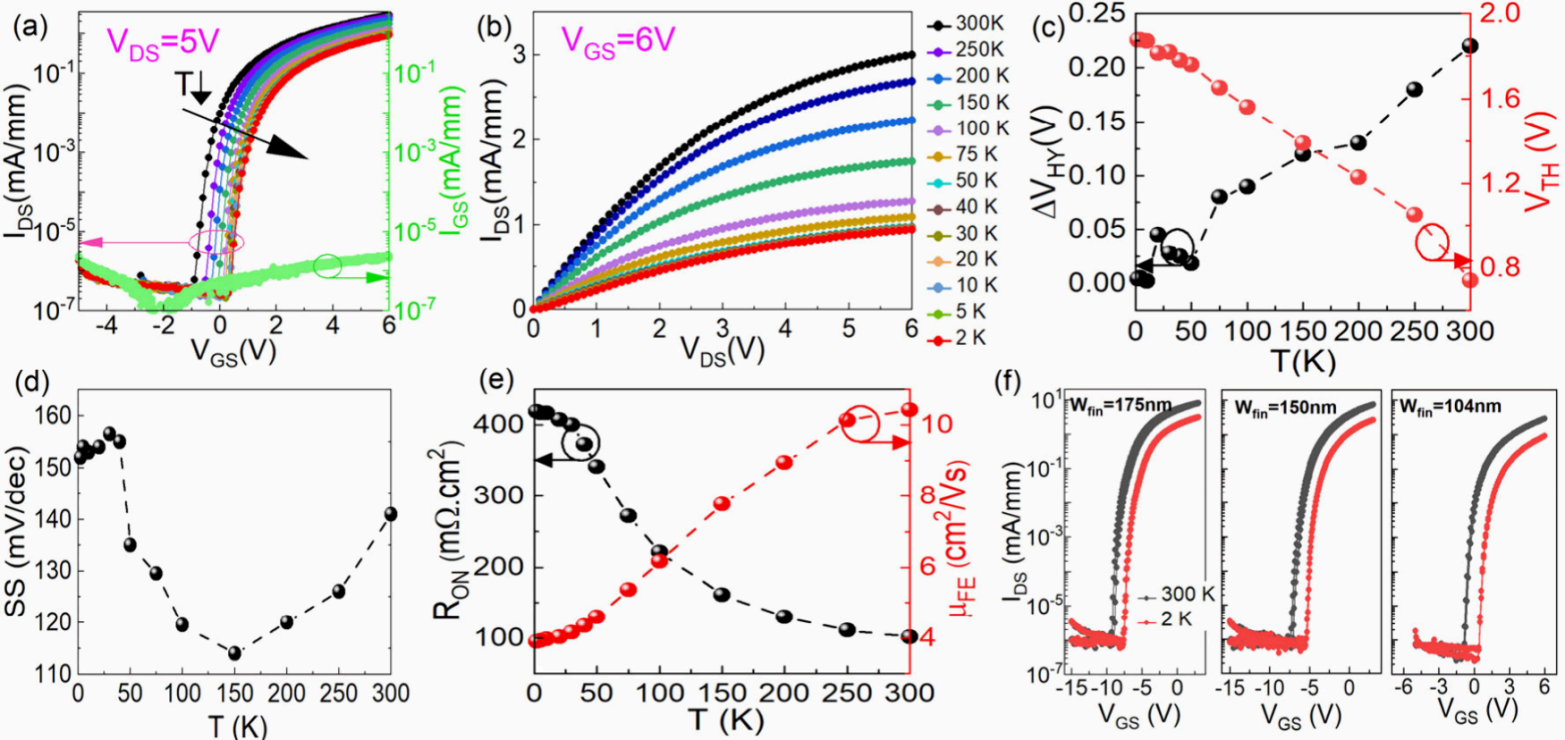
Electrical characterization of β-Ga_2_O_3_ FinFETs at different temperatures from 300 to 2 K: (a) transfer curve at *V*_*DS*_ = 5 V. (b) Output curve at *V*_*GS*_ = 6 V. The behavior of extracted parameters of (c) Δ*V*_*HY*_ and *V*_*TH*_, (d) *SS*, and (e) *R*_*ON*_ and μ_*FE*_. (f) D- and E-mode operation of β-Ga_2_O_3_ FinFETs at 300 and 2 K with three different *W*_*fin*_ values of 175, 150, and 104 nm.

**Figure 3:**
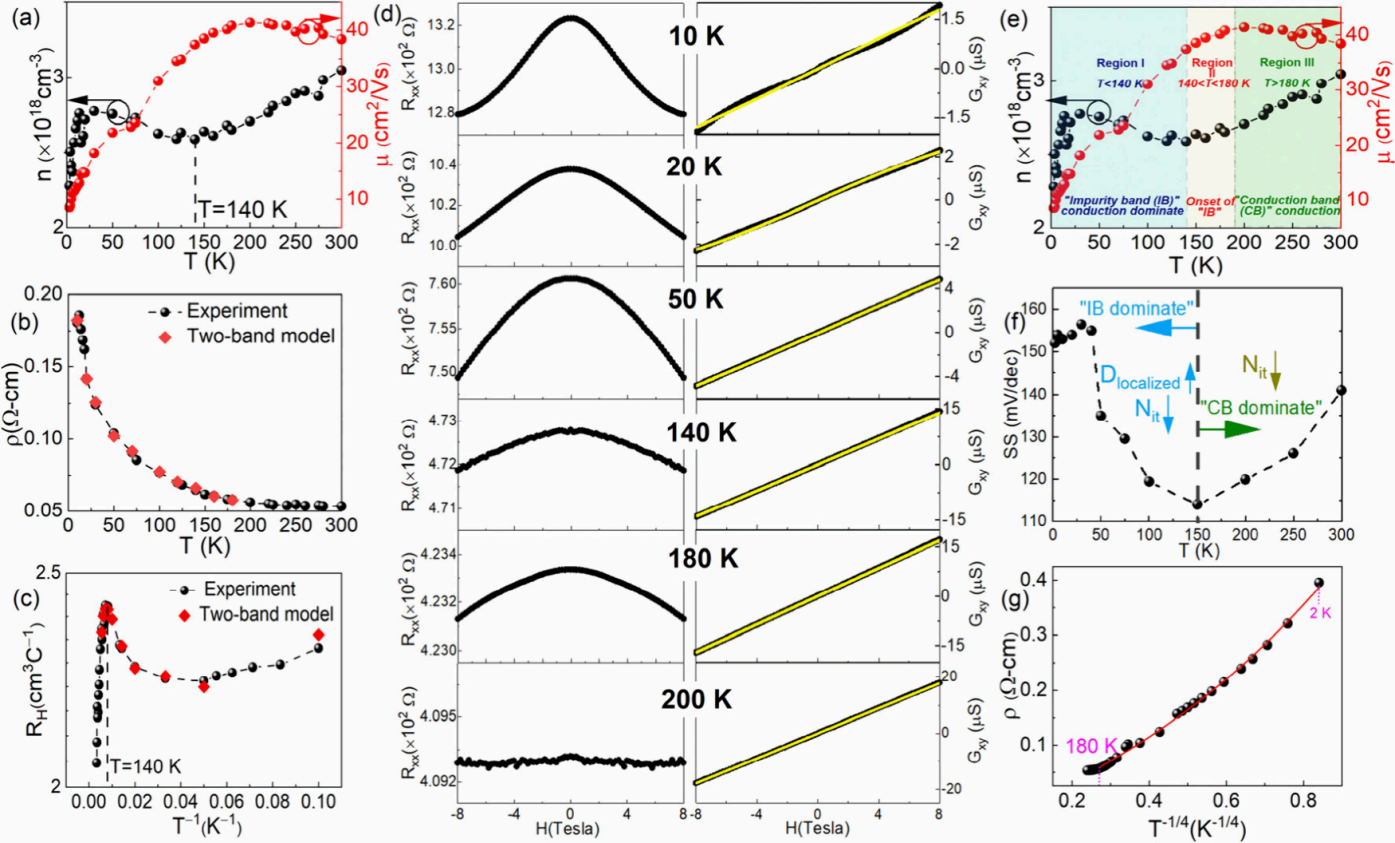
Electrical properties of β-Ga_2_O_3_ films at different temperatures: (a) *n* and μ, highlighting the minimum of *n* at 140 K, (b) ρ, and (c) *R*_*H*_, where the red rhombus in (b) and (c) is from the two-band model, matching exactly with experiments. (d) *R*_*xx*_(*H*), which shows NMR for *T* ≤ 180 K, indicating the presence of IB and *G*_*xy*_(*H*) with a good fitting of the two-band model (yellow solid line). Summary of conduction regions in β-Ga_2_O_3_ films and thereby FinFETs indicating (e) three regions in different ranges of temperatures. (f) Explanation of abnormal behavior of *SS*, attributed to the high density of localized states (*D*_*localized*_) contributed by IB conduction at *T* < 150 K. (g) Mott’s 3D VRH fitting (in red solid line) of the ρ between 2 and 180 K.

Figure 1(h) shows the transfer characteristics of β-Ga_2_O_3_ FinFETs at 300 and 2 K, showing high *I*_*ON*_ */I*_*OFF*_ of 10^7^ and 3 × 10^6^ at a drain–source voltage (*V*_*DS*_) of 5 V with the *V*_*TH*_ of 0.74 and 1.87 V, respectively, from fitting in linear scale, as shown on the right side. The *V*_*TH*_ is positive in both cases, confirming the E-mode operation. The gate–source current (*I*_*GS*_) is ∼10^–6^ mA/mm (noise level) at both 300 and 2 K, confirming a negligible gate leakage current. The *SS*′ of the Ga_2_O_3_ FinFETs are 141 and 152 mV/dec at 300 and 2 K, respectively. Notably, a smaller temperature variation is observed in key device characteristics thanks to the lack of carrier freeze-out in β-Ga_2_O_3_. Relative to 300 K, the *SS* changed by only 11 mV/dec, and the *I*_*OFF*_ remained nearly constant, with a 3.3 times decrease in the *I*_*ON*_ */I*_*OFF*_ ratio at 2 K. This confirms the stable device performance as a function of temperatures. However, a relatively higher shift in *V*_*TH*_ is observed, which is attributed to the freeze-out of interface traps (discussed in Figure 4). This shift can be reduced by achieving a high-quality dielectric–semiconductor interface with low interface trap densities.^26^ The voltage hysteresis (*ΔV*_*HY*_) is 220 mV at 300 K, which is comparable to other β-Ga_2_O_3_ transistors.^27^ It decreases to 4 mV at 2 K, indicating the freeze-out of interface traps at CTs.^26^ The β-Ga_2_O_3_ FinFETs also show stable operation at sub-1 V (Supporting Information, Figure S5), indicating their applicability at low voltages at CTs.

**Figure 4:**
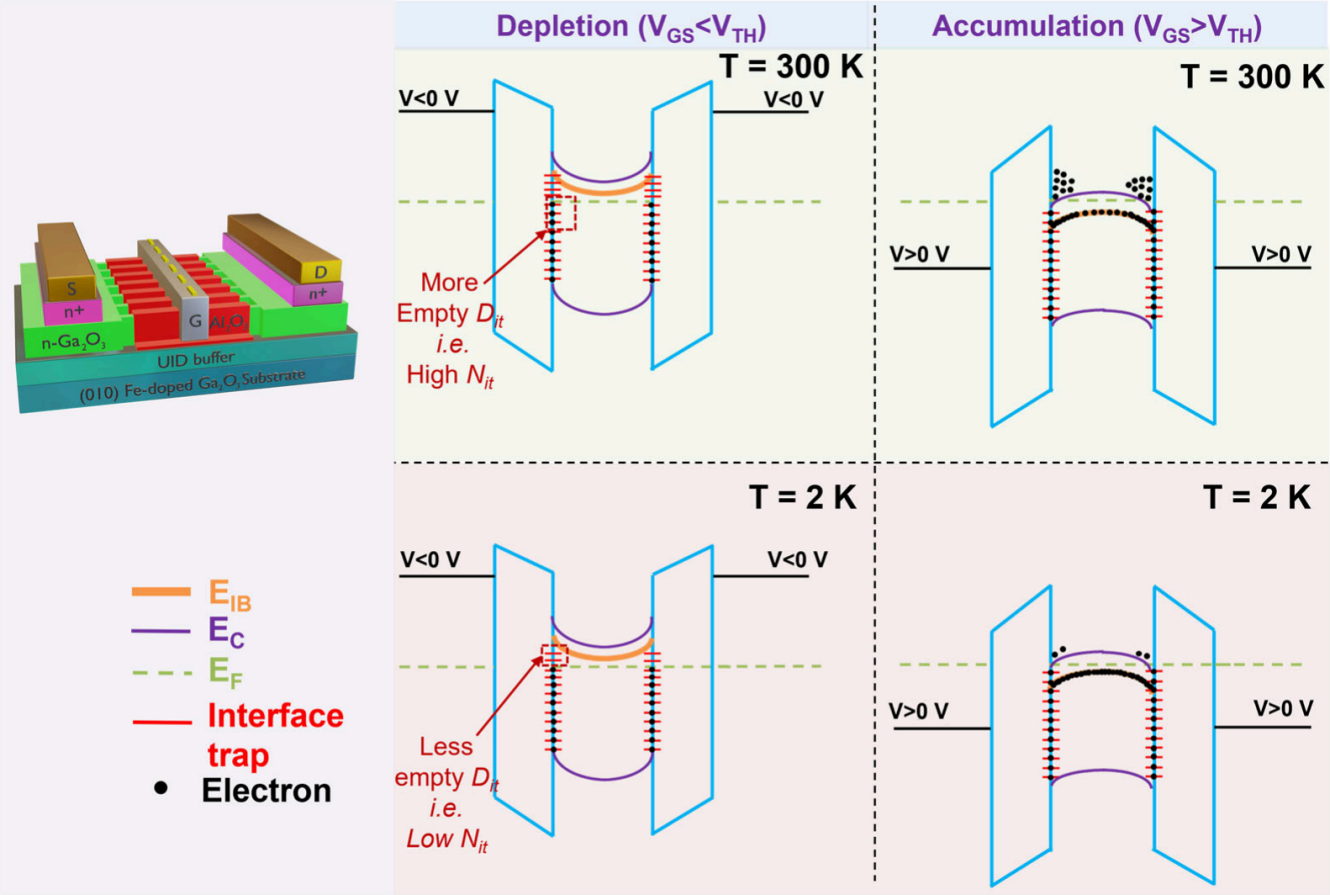
Energy band diagram along the cutline (orange line) shown in the 3D schematic, illustrating the depletion and accumulation regions at 300 and 2 K. More empty *D*_*it*_ and positively charged interface traps charges *N*_*it*_ are observed, while at 2 K, most interface traps are filled with electrons due to freeze-out, resulting in a reduced *N*_*it*_. *E*_*IB*_, *E*_*C*_, and *E*_*F*_ denote the energy levels of the impurity band, conduction band, and Fermi level, respectively.

Figure 1(i) and (j) show the output characteristics of β-Ga_2_O_3_ FinFETs at 300 and 2 K. The maximum drain current (*I*_*DS,max*_) only reduces by ∼3 times at 2 K compared to 300 K at *V*_*GS*_ = 6 V, which is attributed to an increase in *R*_*ON*_ at CTs, resulting from reduced electron mobility at CTs (shown in Figure 3(a)). Additionally, the OFF-state electrical breakdown β-Ga_2_O_3_ FinFETs were measured at room temperature (RT), revealing a breakdown voltage (*V*_*Br*_) of 392 V with a critical electric field of 1.1 MV/cm, shown in Figure S6 (Supporting Information).

To understand the β-Ga_2_O_3_ FinFET operation at CTs, different parameters are extracted in various temperature ranges between 300 and 2 K. Figure 2(a) shows the transfer characteristics of β-Ga_2_O_3_ FinFETs at *V*_*DS*_ = 5 V. A continuous positive *V*_*TH*_ shift is observed with the reduction in *I*_*DS,max*_ (at *V*_*GS*_ = 6 V) by lowering the temperature. The output curve of the β-Ga_2_O_3_ FinFETs at *V*_*GS*_ = 6 V is shown in Figure 2(b), showing the decrease in the on-current identical with the transfer curve. Figure 2(c) shows the trend of *V*_*TH*_ and *ΔV*_*HY*_ with temperature at *V*_*DS*_ = 5 V. The *V*_*TH*_ monotonically shifts positively by 1.13 V, whereas *ΔV*_*HY*_ decreases by 0.22 V with the decrease in temperature. The reduction of *ΔV*_*HY*_ is attributed to the freeze-out of interface traps, further causing the positive *V*_*TH*_ shift, which will be discussed in Figure 4.

Figure 2(d) shows the behavior of *SS* with the temperature. For 150 ≤ *T* ≤ 300 K, *SS* decreases linearly with lowering temperature, following the conventional *SS* equation. However, the *SS* abnormally increases again for temperature *T* < 150 K and reaches 152 mV/dec at 2 K. This unusual behavior of the *SS* may be attributed to another transport mechanism involving electron hopping at CTs,^28^ which is further explained in the discussion of Figure 3(f). Figure 2(e) shows the behavior of *R*_*ON*_ and field effect mobility (*μ*_*FE*_) with the temperature. The *R*_*ON*_ is 100 mΩ·cm^2^ at 300 K, which increases to 415 mΩ·cm^2^ at 2 K, indicating a similar decrease in *I*_*DS,max*_ (Figure 2(a),(b)). The *μ*_*FE*_ is calculated from transconductance at *V*_*DS*_ = 1 V (Supporting Information, Section VII). The value of *μ*_*FE*_ is 10.4 cm^2^/(V s), which decreases by ∼2.5 times with lowering of the temperature.

The precise control over *W*_*fin*_ in FinFETs is crucial to realize depletion (D-) and E-mode operation. A larger *W*_*fin*_ than twice the depletion width will lead to D-mode operation. The underlying mechanism is discussed in the Supporting Information (Section VIII). Figure 2(f) shows the β-Ga_2_O_3_ FinFETs with three different *W*_*fin*_’s of 175, 150, and 104 nm, showing D- and E-mode operations at 300 and 2 K. The β-Ga_2_O_3_ FinFETs with *W*_*fin*_’s of 175 and 150 nm show D-mode operation with a *V*_*TH*_ of −6.55 and −4.42 V at 300 K and −5.03 and −3.27 V at 2 K, respectively, whereas the FinFETs with *W*_*fin*_’s of 104 nm show E-mode operation, as discussed earlier. Note that the D-mode device shows a larger absolute *V*_*TH*_ shift from 300 to 2 K, due to its higher interface trap density, as confirmed by *SS* and *ΔV*_*HY*_ (Section IV, Supporting Information). The variation in device characterization of 10 different E-mode β-Ga_2_O_3_ FinFETs is shown in Figure S3 (Supporting Information).

To understand the principle of electron conduction in β-Ga_2_O_3_ at CTs, a temperature-dependent Hall-effect measurement was performed on an identical Ga_2_O_3_ film used as the FinFET channel. The *n*, μ, Hall-coefficient (*R*_*H*_), and resistivity (ρ) were measured for temperatures ranging from 300 to 2 K. Figure 3(a) shows the behavior of *n* and μ. The value of *n* at 300 K is 3 × 10^18^ cm^–3^, which slightly decreases to 2.5 × 10^18^ cm^–3^ at 140 K and then reaches 2.27 × 10^18^ cm^–3^ at 2 K. The μ first increases due to reduced phonon scattering then monotonically decreases with lowering the temperature and reaches 10.2 cm^2^ V^–1^ s^–1^ at 10 K. Figure 3(b) and (c) show the ρ and *R*_*H*_ with the temperature down to 10 K, revealing two distinct regions: (i) in the temperature range 140 ≤ *T* ≤ 300 K, ρ and *R*_*H*_ both increase with decreasing temperature; (ii) for 10 ≤ *T* ≤ 140 K, ρ continues to increase; however *R*_*H*_ begins to decrease and becomes constant with lowering the temperature. The temperature dependence of *R*_*H*_ explains the behavior of n, explaining an increase in *n* for *T* < 140 K. This behavior can be attributed to two-band conduction where electron conduction occurs through a conduction (CB) and impurity band (IB) at high and low temperatures, respectively.^18^ This crossover of *R*_*H*_ appears at 140 K. Further confirmation of the two-band model is explained with the analysis of temperature-dependent longitudinal (*R*_*xx*_) and Hall resistance (*R*_*xy*_) in the Supporting Information (Section IX).

Figure 3(e) summarizes three conduction regions in the β-Ga_2_O_3_ films based on two-band model analysis: (I) *T* ≥ 200 K: “CB conduction”; (II) 140 ≤ *T* ≤ 180 K: “Onset of IB”; (III) *T* ≤ 140 K: “IB conduction dominates”. Further, the dominance of IB at *T* < 140 K in the films correlates with the observed increase in the *SS* at *T* < 150 K in the β-Ga_2_O_3_ FinFETs. Mott’s equation fitted well (red line) with experimentally measured ρ values as shown in Figure 3(g), which confirms the 3D VRH is the mechanism for electron conduction in IB, similar to those observed in other β-Ga_2_O_3_-doped substrates.^17^

Due to the negligible reduction in the *n* of the channel (Figure 3(a)), it should be noted that the positive *V*_*TH*_ shift in the β-Ga_2_O_3_ FinFETs can be mostly attributed to freeze-out of interface traps like other reports on P-GaN,^8^ SiC,^29^ and Si-based MOSFET^26^ devices. To understand more quantitatively, the *V*_*TH*_ shift can be estimated by the change in positively charged interface state (*D*_*it*_) at 300 and 2 K. The upper limit of the charged *D*_*it*_ can be extracted using the value of *SS* at 300 K, following eq 8.^20^

8 $$D_{it}=\left(\frac{SS\times q}{\ln\left(10\right)\times KT}-1\right)\frac{C_{ox}}{q}$$

The value of *D*_*it*_ at 300 K is ∼1.59 × 10^12^ cm^–2^ eV^–1^. However, it can be ∼10^10^ cm^–2^ eV^–1^ at 2 K, calculated using the correlation between occupation of *D*_*it*_ and *ΔV*_*HY*__,_ particularly at CTs, discussed in the Supporting Information (Section XVI). So, the overall reduction of *D*_*it*_ from 300 to 2 K will be ∼1.5 × 10^12^ cm^–2^ eV^–1^, leading to the positive shift of *V*_*TH*_ by ∼1.1 V.^30^ This matches exactly with the total positive *V*_*TH*_ shift of β-Ga_2_O_3_ FinFETs, confirming the contribution of interface trap freeze-out. Further calculation details along with UV-assisted experiments can be found in the Supporting Information (Sections XVI, XVII).

Due to the absence of high-performance p-channel β-Ga_2_O_3_, it is challenging to realize the homogeneous β-Ga_2_O_3_ CMOS ICs. However, it is possible to fabricate β-Ga_2_O_3_ NMOS ICs using monolithic integrated D- and E-mode FETs, which can be utilized as load and driver, respectively, with a driver-to-load resistance ratio (α) of ∼50. A higher positive *V*_*TH*_ of 2.55 V (at 300 K) for the FinFET is employed for the driver transistor to ensure the circuit operation between the 0 and *V*_*dd*_ input voltages. Further details are discussed in the Supporting Information (Section XVIII). Figure 5 depicts the characteristics of the β-Ga_2_O_3_ logic inverter ICs at 300 and 2 K. The voltage transfer characteristics (*V*_*TC*_) of the inverter, as illustrated in Figure 5(a), reveal a high-level output voltage (*V*_*OH*_) of 4.93 and 4.90 V, with a low-level output voltage (*V*_*OL*_) of 0.003 and 0.02 V, respectively, resulting in a large voltage swing of about 98.5% (4.927 V/5 V) and 97.6% (4.88 V/5 V) at 300 and 2 K, respectively. The right shift in the *V*_*TC*_ curve is associated with the positive shift in transfer characteristics induced by the freezing of the interface traps, as discussed earlier. Figure 5(b) shows the voltage gain of 31 and 28 at 300 and 2 K, nearly constant with temperature, attributed to the identical value of α, irrespective of temperature. Further, the static power dissipation (*P*_*static*_) of the β-Ga_2_O_3_ inverter was calculated, depicting ∼0.88 and ∼0.13 μW at 300 and 2 K in Figure 5(c). The peak value of *P*_*static*_ for the β-Ga_2_O_3_ inverter at 300 K is comparable to the GaN CMOS inverter IC.^31^ The low- and high-level input transition voltages (*V*_*IL*_ and *V*_*IH*_) were defined at unit-gain points, shown with purple lines in Figure 5(d), which further leads to the low- and high-noise margins (*NM*_*L*_ and *NM*_*H*_) (yellow shaded area) and transition voltage regions (green shaded region). Figure 5(e) shows the *V*_*TC*_ at various temperatures down to 2 K. The extracted parameters such as logic ‘low’ and ‘high’ output voltages (*V*_*OL*_ and *V*_*OH*_, respectively), *V*_*IL*_, *V*_*IH*_, and *V*_*TH*_ are depicted in Figure 5(f) at different temperatures.

**Figure 5:**
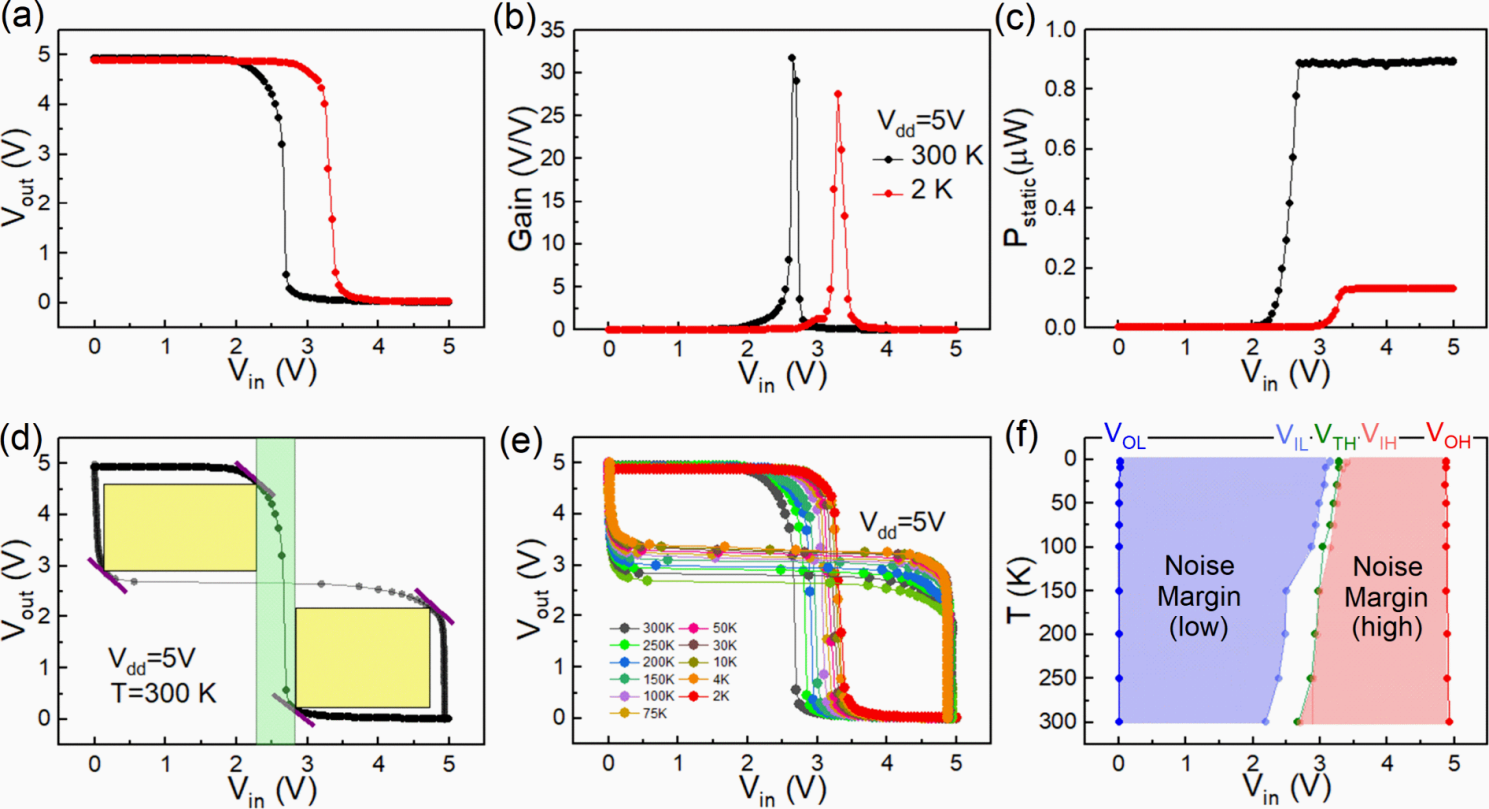
Electrical characterization of a monolithically integrated Ga_2_O_3_ NMOS inverter at *V*_*dd*_ of 5 V: (a) *V*_*TC*_, (b) voltage gain. (c) *P*_*static*_ at 300 and 2 K, respectively. (d) Noise margins (yellow shaded areas) at *V*_*dd*_ of 5 V at 300 K with transition window (green shaded area). (e) *V*_*TC*_ at various temperatures down to 2 K at *V*_*dd*_ = 5 V. (f) The behavior of extracted parameters *V*_*OL*_, *V*_*IL*_, *V*_*TH*_, *V*_*IH*_, and *V*_*OH*_ at different temperatures.

Figure S14­(a) and (b) show the benchmarking of *I*_*ON*_ */I*_*OFF*_ and *SS* of our D- and E-mode Ga_2_O_3_ FinFET with other Ga_2_O_3_ FinFETs at CTs. Both D- and E-mode Ga_2_O_3_ FinFETs show relatively higher *I*_*ON*_ */I*_*OFF*_ and lower *SS* at 300 K, compared to other literature, whereas, at 2 K operation, this is the first demonstration with Ga_2_O_3_ technology.

In summary, this work demonstrated the operation of UWBG β-Ga_2_O_3_ FinFETs and logic inverter ICs at CTs down to 2 K. The FinFETs exhibit E-mode operation with stable device characteristics including *SS*, *I*_*OFF*_, *I*_*ON*_ */I*_*OFF*_, etc., as a function of temperature thanks to the lack of carrier freeze-out. Based on the two-band model, it is concluded that the electron conduction through IB induced by Si doping impurities is the major reason behind CT operation for *T* < 150 K with Mott’s 3D VRH as the mechanism of electron conduction. Moreover, the monolithically integrated Ga_2_O_3_ NMOS ICs indicate their applicability for cryogenic logic chips. This work represents the initial demonstration of the high-performance UWBG Ga_2_O_3_ electronics for cryogenic operation, making a significant development in the field of cryogenics and, thereby, extreme temperature electronics. Further, it paves the way for a new opportunity for other types of Ga_2_O_3_ technology using MOCVD and MBE grown films for power and RF devices, photodetectors, memory, etc., to realize highly integrated and compact cryogenic and extreme-temperature electronics.

## Supplementary Material



## Data Availability

The data that support the findings of this study are available from the corresponding author upon reasonable request.

## Notes

Details of substrate cleaning and PLD growth of UID and Si-doped β-Ga_2_O_3_; methods of FinFET fabrication; TEM/EELS structural analysis; and cryogenic electrical setups (PPMS + Keithley) for transistor and inverter testing; key electrical characteristics, physics models, two-band Hall/conductance analysis, 3D Mott VRH models, discussion of cryogenic *V*_*TH*_ shifts, hysteresis freeze-out, *SS* increase, UV-assisted detrap experiments, statistical device variation, and inverter design/benchmarking (PDF)
