# Supplementary material for: Two Kelvin Operation of Ultrawide-Bandgap β‑Ga2O3 FinFETs and Logic Inverter Integrated Circuits
Source: Nano Lett. 2026 Feb 25;26(17):5660–7. doi: 10.1021/acs.nanolett.5c06155 (PMC13154364; doi:10.1021/acs.nanolett.5c06155)
Supplement: Supplementary file 1 [file nl5c06155_si_001.pdf]

Supporting Information for
2 Kelvin Operation of Ultrawide Bandgap β-Ga_2O_3 FinFETs and Logic Inverter Integrated Circuits

Vishal Khandelwal[1], Glen Isaac Maciel García[1], Mritunjay Kumar[1], Francesco Blanda[1], Na Xiao[1], Dongxing Zheng[2], Ganesh Mainali[1], Manoj Kumar Rajbhar[1], Xiao Tang[1], Xixiang Zhang[2], Xiaohang Li[1]*

[1]Advanced Semiconductor Laboratory, Electrical and Computer Engineering Program, Computer, Electrical, and Mathematical Sciences and Engineering (CEMSE), King Abdullah University of Science and Technology (KAUST), Thuwal 23955-6900, Kingdom of Saudi Arabia.

[2]Experimental Spintronics & Low Dimensional Materials and Physics Laboratory, Material Science and Engineering, Physical Science and Engineering Division (PSE), King Abdullah University of Science and Technology (KAUST), Thuwal 23955-6900, Kingdom of Saudi Arabia.

*Corresponding author: xiaohang.li@kaust.edu.sa

Section I. Advantages of FinFETs Architecture for Cryogenic Operation

1. β-Ga_2O_3 FinFETs for Stable, High-Performance Cryogenic Operation

Three-dimensional gate control of β-Ga_2O_3 FinFETs provides superior stability and temperature independence at CTs compared to planar transistors. In contrast, planar enhancement-mode (*E*-mode) transistors rely on either thinning the channel or etching

recess structures, which can increase source-drain resistance, lower on-current, and enhance interface trap density, leading to instability and threshold voltage shifts at CTs. Thanks to high aspect ratio Fin structures, a high current on-off ratio can be obtained. Further, an atomically smooth MOS interface can avoid the instability in transistor characteristics at CTs.

Additionally, a moderately high electron concentration (~10^{18} cm^{-3}) is necessary to prevent carrier freeze-out in β-Ga_2O_3 films. This can be effectively met by the FinFET design by enhancing gate control to realize high-performance E-mode operation at CTs. Further, the FinFET gate forms the depletion region from multiple sides as shown energy band diagram (Figure 4) and supplementary material (Figure S3), which can allow a thicker channel and thereby high on-off current ratio.

2. **Seamless on-chip integrated β-Ga_2O_3 Logic with FinFET Architecture**

As discussed in the manuscript, integrating both Depletion-mode (D-mode) and E-mode transistors is essential for compact, high-performance cryogenic circuits like logic gates and SRAM devices. The FinFET structure uniquely enables both types of transistors on the same wafer simply by tuning the fin width in FinFETs, as shown in Figure 2(f)—achieved through precise lithography without additional processing steps, unlike planar designs.

3. **β-Ga_2O_3 FinFETs for Miniaturized Cryogenic Circuits**

Modern ultra-scaled Si cryogenic devices rely on FinFET technology for enhanced gate control, energy efficiency, and high-density integration. β-Ga_2O_3 FinFETs employed in this study can achieve similar scalability and adaptability for cryogenic logic circuits, making them viable for high-end, miniaturized cryogenic systems.

Section II. Calculation of channel width (W_{CH})

The channel width (W_{CH}) of the FinFET is defined as = Total no. of fins × (2 × Fin height + Fin width). The Fin height is ~ 600 nm in the β-Ga_2O_3 FinFET.

So, the W_{CH} = 55 × (2 × 600 nm + 104 nm) ~ 70 μm

Section III.

Experimental Section

Substrate Cleaning and Preparation

First, the semi-insulating Fe-doped Ga_2O_3 (010) substrates (Novel crystal technology, Japan) were cleaned with Acetone, 2-propanol, and DI water for 5 minutes using ultrasonication. Subsequently, the substrates were immersed at room temperature in the Piranha (H_2SO_4:H_2O_2 = 3:1) solution for 10 minutes followed by Hydrofluoric (HF) acid for 30 minutes to avoid the Si parasitic.

Homoepitaxial β-Ga_2O_3 Growth

The cleaned substrates were immediately transferred into the Neocera pulsed laser deposition (PLD) chamber for Ga_2O_3 growth. First, samples were in-situ annealed at 600 °C in O_2 ambience for 30 minutes, followed by the growth of 250 nm un-intentional doped (UID) Ga_2O_3 layer at 650 °C. After this, a 600 nm of n-Ga_2O_3 film is grown at 700 °C in the O_2/Ar = 2/98 sccm ambient. These growths were employed by ablation of undoped and Si-doped 1-inch targets, respectively. For the Si-doped Ga_2O_3 target, the Si atomic percent was 0.025 at%, which leads to Si doping density of ~ 5×10^{18} cm^{-3}. The chamber pressure of 10 mTorr, laser energy of 100 mJ, and laser frequency of 5 Hz were fixed during the deposition. The thickness of the film is confirmed by

realizing the same growth on a Sapphire substrate and then measured using the reflectance measurement.

Characterization of *β*-Ga_2O_3 Thin Films

(a) Crystalline and Surface Characterization

The rocking curve full-width half maxima (RC-FWHM) of as-grown Ga_2O_3 film was measured using Bruker D8 ultra-X-ray diffraction tool. The surface roughness of the film was determined using atomic force microscopy (AFM) from Bruker Dimension Icon.

(b) Longitudinal and Hall Magnetoresistance Measurement

For this, the Ti/Au (20/150 nm) metal is deposited on the four corners of a Ga_2O_3 thin film sample. The measurement is performed in the presence of a high magnetic field between -8 to 8 Tesla at different temperatures down to 2 K. Both measurements are done using the Physical property measurement system (PPMS) in the VanderPauw configuration. Room temperature Hall-effect measurement was performed to determine the carrier concentration and mobility of n-Ga_2O_3 using the Ecopia HMS 3000 system in the Vander-Pauw configuration with the magnetic field of 0.525 Tesla.

Fabrication of *β*-Ga_2O_3 FinFET

The key steps of fabrication of Ga_2O_3 FinFET are shown in the supplementary material (Figure S11). Device fabrication started with patterning the nanometer-scaled fins using Crestec electron beam lithography (EBL) and deposition of 120 nm Ni mask using electron beam evaporator followed by metal-liftoff. Subsequently, the BCl_3/Ar dry etching was performed to define the Mesa with a height of ~ 600 nm thickness followed by removal of residual Ni using wet etchant. Afterward, the sample was immersed in diluted HF (1:1) for 10 minutes to facilitate the recovery

of dry-etch-induced surface damage. A 200 nm thick SiO_2 is deposited using Plasma enhanced chemical vapor deposition (PECVD) at 300 °C and then patterned the source-drain (SD) region using conventional lithography. Then the SD area is opened from SiO_2 using Buffer-oxide etchant (BOE) dip (1.5 min) for further growth of ~120 nm of n+-Ga_2O_3 at 700 °C using PLD. The n+-Ga_2O_3 is grown in the SD region to ensure the low contact resistance of the device. Further, the sample is immersed in HF for 1 min to remove the SiO_2 and Ga_2O_3 from areas other than SD. Later, a 25 nm of Al_2O_3 gate dielectric is deposited using the atomic layer deposition (ALD) at 250 °C temperature followed by selectively dry etching with BCl_3/Ar, outside the fin region. Further, the SD area is patterned with conventional lithography to deposit 20/150 nm thick Ti/Au using sputtering, and then, it is annealed in N_2 at 450 °C for 70 Sec. This annealing helps to create low contact resistance as well as a good dielectric/semiconductor interface. Finally, a 100/100 nm of Pt/Au was deposited with the same process as SD to serve as gate metal.

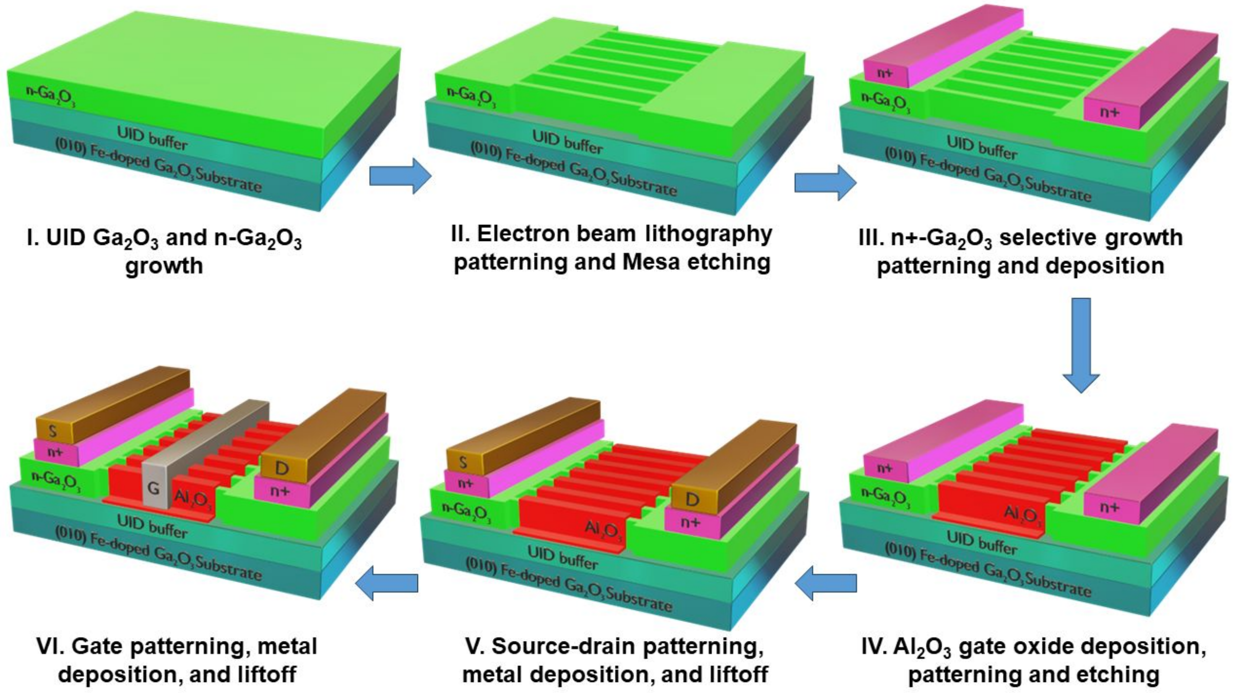

Figure S1. Fabrication process flow of β-Ga_2O_3 FinFETs, explaining the key process steps.

Characterization of *β*-Ga_2O_3 FinFET

(a) TEM and EELS measurement

A TEM lamella was prepared using Helios G4 (Thermo Fisher Scientific). First, a protection layer was deposited using the electron-beam and Ga ion beam assisted Pt deposition at the interested device region. After that, bulk milling and in situ lift-out is carried out to transfer the lamella to an Omniprobe TEM grid. The thinning of the TEM lamella is performed using a 30 kV Ga ion beam down to around 150 nm in thickness, followed by the low-kv cleaning using 5 kV and 2 kV Ga ion beam to remove the damaged layer during the milling. Scanning transmission electron microscopy (STEM) is carried out using a double Cs corrected Themis Z (Thermo Fisher Scientific Inc.) operated at 300 kV. The convergence semi-angle of the electron probe and the collection semi-angle of the high angle annular dark-field (HAADF) image is 21 mrad and 49-200 mrad, respectively. Elemental mapping is carried out using electron energy loss spectroscopy (EELS) spectrum imaging technique on a Gatan Continuum with K3 camera. The collection semi-angle of EELS is around 75 mrad.

(b) Set-up for transistor and inverter IC measurement at Cryogenic temperature (CT)

The electrical characteristics of the transistor and inverter IC were measured by utilizing the Quantum design Dynacool PPMS and Keithley 4200A semiconductor parameter analyzer. The PPMS was used to achieve the CT down to 2 K whereas the Keithley 4200A for electrical measurement. First, the sample was wire bonded on the PPMS sample holder (Puck) and then loaded into the PPMS chamber. After this, a specially designed connection box was used to connect the PPMS and Triax cables of the Keithley 4200 A. For measuring the characteristics of

the transistor and inverter IC at different temperatures, first, the temperature was changed using the PPMS controller computer manually and then measurement was done using the Keithley 4200A system. The schematic of the cryogenic-temperature transistor and inverter IC measurement set-up is explained in following Figure S.

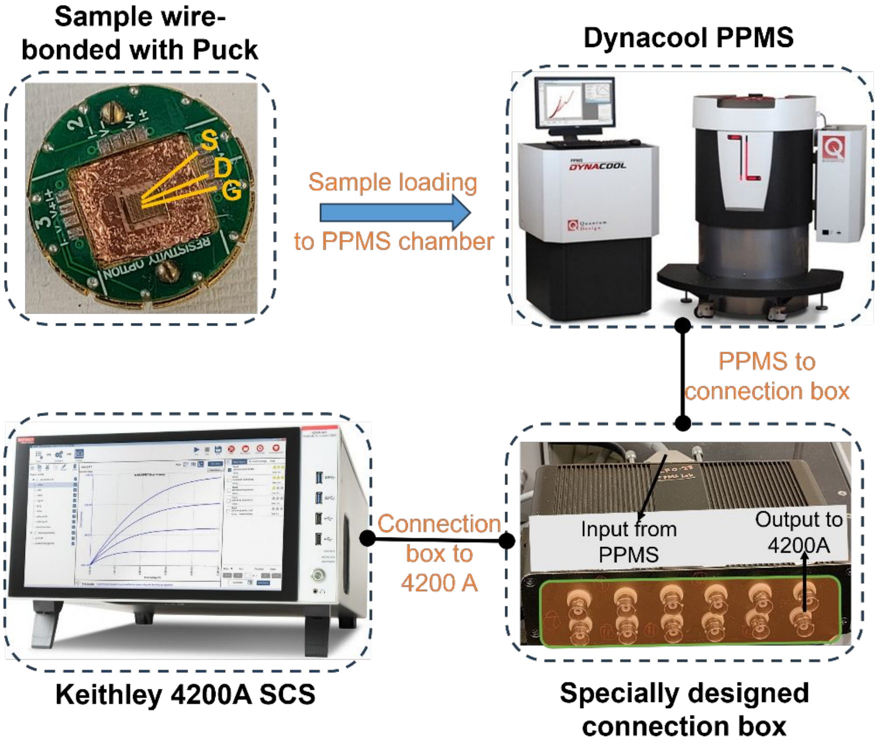

Figure S2. Schematic of the measurement setup for FinFETs and inverter ICs.

(c) High voltage (electrical breakdown) measurement

High voltage measurement of Ga_2O_3 FinFET is performed using Keysight B1505A power device analyzer at RT.

Statistical Analysis

To study the variation in the device performance of E-mode FinFETs, ten devices are characterized both at 300 and 2 K on the same wafer. Figure S3 shows the variation in V_{TH}, I_{ON}/I_{OFF}, ΔV_{HY}, and

SS of the 10 different β-Ga_2O_3 FinFETs at 300 and 2 K. The V_{TH} and I_{ON}/I_{OFF} show relatively larger variations than the ΔV_{HY} and SS, which can be further improved by controlling the variation in the Fin width of FinFET devices.

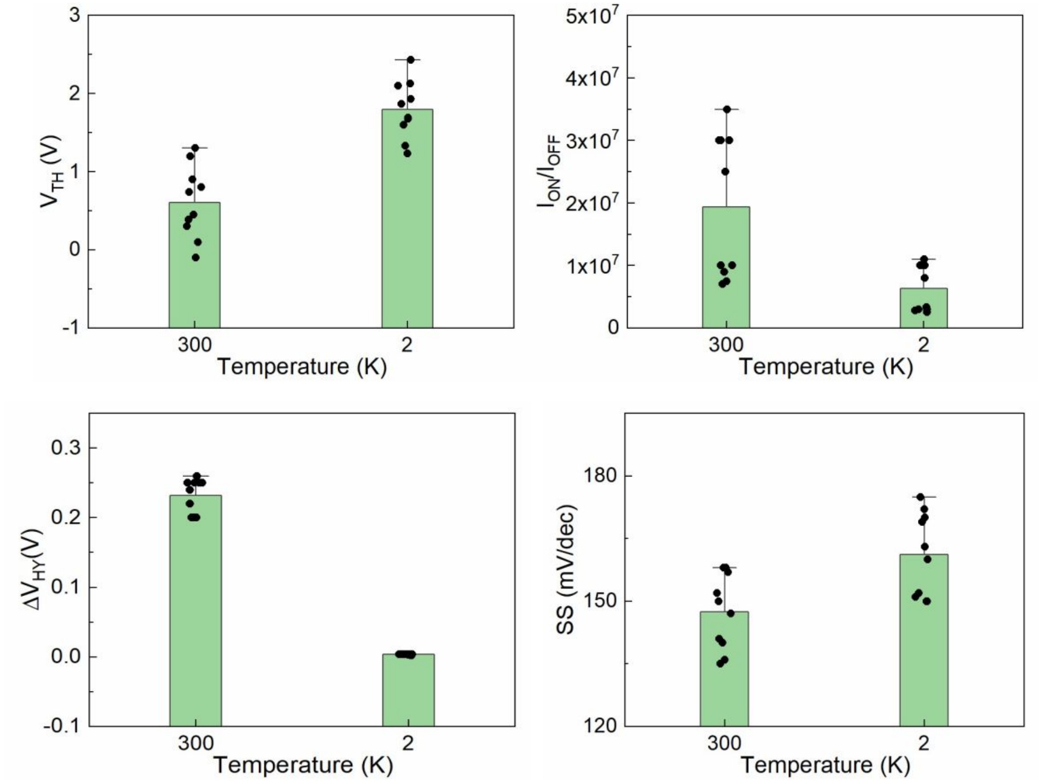

Figure S3. Electrical characterization of ten FinFETs, showing the variation in V_{TH}, I_{ON}/I_{OFF}, ΔV_{HY}, and SS at 300 and 2 K.

Section IV. Depletion-mode vs Enhancement-mode Device: Discussion on FinFET Interface

High-aspect-ratio fins in β-Ga_2O_3 FinFETs are typically defined using dry plasma etching, which can introduce sidewall damage in the form of dangling bonds, defect states, and increased surface roughness. These effects can lead to elevated dielectric–semiconductor interface trap density (D_{it}), resulting in degraded electrical characteristics such as reduced on-current, increased subthreshold swing (SS), enhanced surface scattering, and pronounced voltage hysteresis (ΔV_{HY})[1]. To mitigate

such damage, the fins in this work were treated with a diluted HF solution (1:1) for 10 minutes, which facilitates partial recovery of dry-etch-induced surface damage and improves sidewall quality.

The impact of plasma-etch-induced sidewall damage and the associated interface traps on FinFET behavior at room and cryogenic temperatures can be understood by comparing two FinFET devices with different D_{it}, fabricated with fin widths (W_{fin}) of 104 nm and 175 nm, respectively, as shown in Fig. 2(f) of the manuscript.

At room temperature (300 K), it is observed that the D-mode device exhibits both a larger *SS* and greater voltage hysteresis (ΔV_{HY}) compared to the E-mode device indicating a higher interface trap density. Quantitatively, the SS values are 200 mV/dec and 141 mV/dec for D-mode and E-mode devices respectively at 300 K, corresponding to extracted D_{it} of 2.27×10^{12} cm^{-2} eV^{-1} and 1.59×10^{12} cm^{-2} eV^{-1}, respectively, calculated using Equation 8 of the manuscript. This difference can be attributed to (i) the larger dielectric–semiconductor interface area associated with the wider fin in the D-mode device and (ii) the higher absolute carrier concentration in D-mode operation, which increases trapping and de-trapping activity at interface states.

Therefore, comparing these devices can indicate the impact of D_{it} on transistor characteristics at room and cryogenic temperatures.

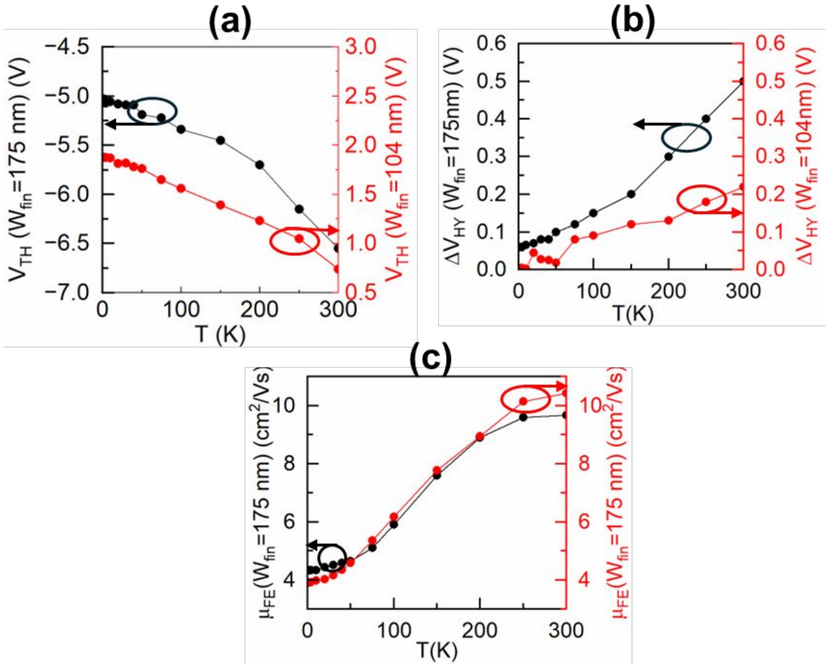

Figure S4. The trend of the key device parameters, threshold voltage (V_{TH}), voltage hysteresis (ΔV_{HY}), and field-effect mobility (μ_{FE}) for D-mode and E-mode devices.

Figure S4 shows the key device parameters, threshold voltage (V_{TH}), voltage hysteresis (ΔV_{HY}), and field-effect mobility (μ_{FE}) at cryogenic temperatures (CTs).

1. $\boldsymbol{V_{TH}}$: Due to higher D_{it} in the D-mode device, it shows a larger V_{TH} shift of ~2 V compared to the E-mode device of ~1.13 V (shown in Figure S4(a)), which is consistent with the discussed analysis of the correlation of V_{TH} with D_{it} in the manuscript.
2. $\boldsymbol{\Delta V_{HY}}$: D-mode shows a large ΔV_{HY} of 0.5 V at 300 K, compared to E-mode in Figure S4(b), which shows 0.22 V, attributed to higher D_{it}. At CTs, both show a reduction in ΔV_{HY}, due to freeze-out of interface traps.
3. $\boldsymbol{\mu_{FE}}$: Interestingly, both devices show a similar trend at CTs as shown in Figure S4(c). Typically, the higher D_{it} value should show lower μ_{FE} in D-mode devices, but it can be counteracted by the large channel width, which may

have lower surface scattering than in E-mode devices, where it may be dominant.

Section V. Sub-1 V operation voltage characterization

β-Ga_2O_3 FinFETs offer flexibility in operation, allowing for both sub-1V and high-voltage performance at CTs. To show the FinFET operation at sub-1V, we characterized the device down to 2 K at a low drain voltage-source voltage (V_{DS}) of 0.75 V, shown in Figure S5.

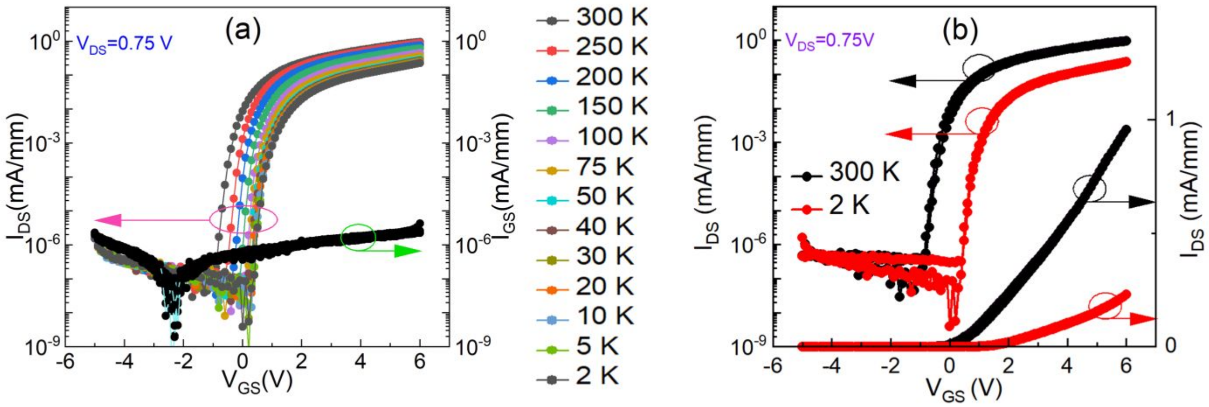

Figure S5. Transfer characteristics of β-Ga_2O_3 FinFET at V_{DS} of 0.75 V at (a) different temperatures between 300 to 2 K, (b) 300 and 2 K along with hysteresis (right y axis show linear scale).

Figure S5(a) presents the transfer characteristics of the β-Ga_2O_3 FinFET across various temperatures down to 2 K. All key device parameters, including threshold voltage, subthreshold swing (*SS*), and hysteresis, remain consistent at a V_{DS} of 5 V. Notably, the hysteresis voltage is approximately 0.11 V at 300 K and reduces to ~ 0.001 V at 2 K, as shown in Figure S5(b) due to interface trap freeze-out.

Section VI. Electrical breakdown measurement of β-Ga_2O_3 FinFETs

Figure S6 shows the electrical breakdown of β-Ga_2O_3 FinFETs at room temperature (RT), showing breakdown voltage (V_{Br}) = 392 V. This leads to average critical electric field ($E_{critical}$) = 392 V/3.5 μm (V_{Br}/L_{GD}) = 1.12 MV/cm.

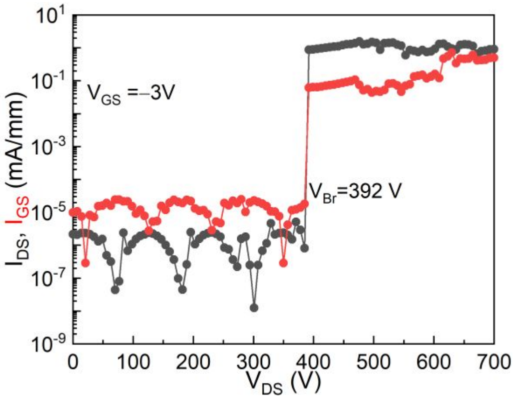

Figure S6. Electrical breakdown measurement of β-Ga_2O_3 FinFETs at RT.

Section VII. Calculation of field effect mobility

The field effect mobility (μ_{FE}) is calculated using Equation (S1), as follows.

$$\mu_{FE} = \frac{L_{CH}\, g_m}{W_{CH} C_{ox} V_{DS}} \qquad \text{(S1)}$$

where, L_{CH} is the FinFET channel length = 10 μm, W_{CH} is the FinFET channel width = 70 μm, C_{ox} is dielectric (Al_2O_3) capacitance = 2.124×10^{-7} F/cm^2, V_{DS} is drain-source voltage = 1 V, $g_m = \frac{\partial I_{DS}}{\partial V_{GS}}$ is the transconductance at V_{DS} of 1 V (linear region) extracted from the transfer curve at different temperatures.

Section VIII. Underlying Mechanism Delpletion Mode FinFET with Fin Width

A FinFET with a fin width (W_{fin}) larger than the depletion width leads to depletion-mode (*D*-mode) operation, which can be explained using a one-dimensional depletion approximation and the underlying band diagram. In NMOS transistors, depletion-mode (*D*-mode) and enhancement-

mode (E-mode) devices are characterized by negative and positive threshold voltages (V_{TH}), respectively. These correspond to normally-ON and normally-OFF devices at zero gate-to-source voltage ($V_{GS} = 0$ V).

For a junctionless n-channel transistor, the OFF and ON states at $V_{GS} = 0$ V are governed by the depletion and accumulation of electrons in the channel beneath the gate. The OFF state requires complete depletion of electrons in the channel region under the gate, thereby preventing current flow between source and drain. If the electrons are not fully depleted, the device remains ON at zero gate bias.

The depletion region at $V_{GS} = 0$ V can be quantified by the depletion width (W_{DG}) under the gate. This width can be estimated using the depletion approximation as Equation (S2)[2]:

$$W_{DG} = \left[\frac{2\epsilon_0\epsilon_r V_{FB}}{qN_d}\right]^{\frac{1}{2}} \qquad \text{(S2)}$$

where ϵ_0 and ϵ_r are the vacuum and relative permittivity, respectively; N_d is the electron carrier concentration; V_{FB} is the flat band voltage.

The flat band voltage V_{FB} is determined using the Equation (S3).

$$V_{FB} = \phi_{ms} + \frac{Q_{it}}{C_{ox}} \quad \text{(S3)}$$

Similarly, the depletion depth in the ungated region W_{D-UG} is given by Equation (S4).

$$W_{D-UG} = \frac{Q_{it}}{N_D} \quad \text{(S4)}$$

Thus, the depletion width depends on interface properties such as ϕ_{ms} and Q_{it}, which are related to the MOS interface and metal stack.

In FinFET devices, the gate controls the channel from three sides, as illustrated in Figure 1(g) (main manuscript), where the gate wraps around the fin. The depletion region forms from both sides of the fin channel due to ϕ_{ms} and Q_{it}. Figure S7 shows the 2D schematic diagram of the FinFET channel, with the gate shown on both sides of Fin, controlled vertically from current conduction.

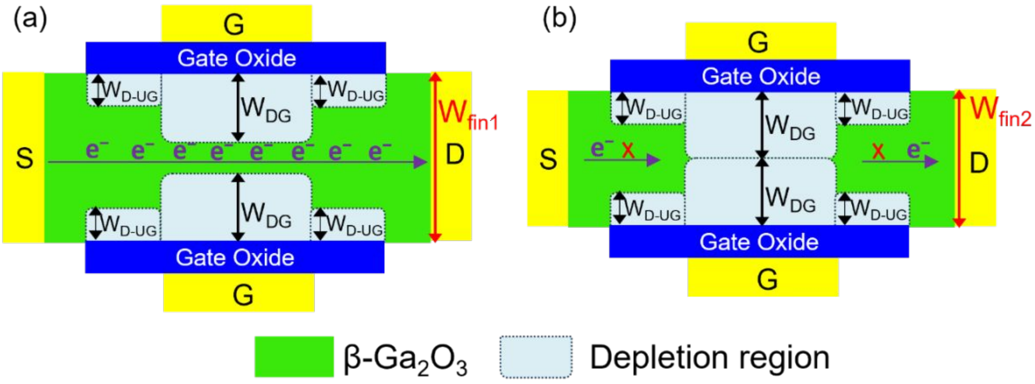

Figure S7. Two-dimensional schematic of Fin structures illustrating the depletion regions in both gated (W_{DG}) and ungated ($W_{D\text{-}UG}$) areas for (a) depletion-mode (*D*-mode) and (b) enhancement-mode (*E*-mode) FinFETs. An electron conduction path exists when the fin width, $W_{fin} > 2\times W_{DG}$, resulting in a D-mode device. Conversely, when $W_{fin} \leq 2\times W_{DG}$, the conduction path is fully depleted, resulting in an E-mode device. All W_{DG} and $W_{D\text{-}UG}$ are defined at $V_{GS} = 0$ V.

When W_{fin} is larger than twice the depletion width W_{DG}, the depletion regions from the two sides cannot fully overlap, leaving an undepleted channel region that conducts current at zero gate bias (Figure S7(a)). This results in a normally ON device, i.e., depletion-mode operation, requiring a negative gate voltage to fully deplete the channel and turn OFF the device.

Conversely, when W_{fin} is smaller or comparable to twice the W_{DG}, the depletion regions from both sides overlap and fully deplete the channel at zero gate bias (Figure S7(b)). In this case, a positive

gate voltage is needed to accumulate electrons and turn the device ON, exhibiting enhancement-mode behavior.

Section IX. Principle of cryogenic operation of β-Ga_2O_3 FinFET

Considering the two-band model, the ρ and R_H can be defined as the following Equations (1) and (2)[3].

$$\rho = \frac{t}{[n_1\mu_1+n_2\mu_2]e} \quad (1)$$

$$R_H = \frac{[n_1\mu_1^2+n_2\mu_2^2]}{e[(n_1\mu_1+n_2\mu_2)^2]} \quad (2)$$

where (n_1, μ_1), (n_2, μ_2) are sheet carrier concentration and bulk mobility in CB and IB and t is the thickness of film.

To confirm the presence of two bands (CB and IB) in the β-Ga_2O_3 films and thereby FinFETs, Equations (1) and (2) must satisfy the experimentally observed ρ and R_H, which require the value of (n_1, μ_1), and (n_2, μ_2). Therefore, temperature-dependent longitudinal (R_{xx}) and Hall resistance (R_{xy}) were measured and analyzed with the two-band model where $R_{xy}(H)$ can be written as Equation (3)**Error! Bookmark not defined.**.

$$R_{xy}(H) = \left[\frac{\left(-\frac{H}{e}\right)[(n_1\mu_1^2+n_2\mu_2^2)+H^2\mu_1^2\mu_2^2\,(n_1+n_2)]}{[(n_1\mu_1+n_2\mu_2)^2+H^2\mu_1^2\mu_2^2\,(n_1+n_2)^2]}\right] \quad (3)$$

Equation (3) has four unknowns (n_1, μ_1,n_2, and μ_2) which can be further reduced to two (μ_1, μ_2) by introducing the Hall conductance $G_{xy}\,(H)$, calculated using experimentally measured R_{xy} and R_{xx} as following Equation (4).

$$G_{xy} = -\frac{R_{xy}}{\left[R_{xy}^2 + R_{xx}^2\right]}$$

$$= eH\left[\frac{(C_1\mu_1 - C_2)}{\left(\frac{\mu_1}{\mu_2} - 1\right)(1 + \mu_2^2 H^2)} + \frac{(C_1\mu_2 - C_2)}{\left(\frac{\mu_2}{\mu_1} - 1\right)(1 + \mu_1^2 H^2)}\right] \quad (4)$$

where

$$C_1 = n_1\mu_1 + n_2\mu_2 \quad (5)$$

$$C_2 = n_1\mu_1^2 + n_2\mu_2^2 \quad (6)$$

Note that C_1,C_2 can also be measured experimentally. $C_1 = \frac{G_{xx}(0)}{e}$ where $G_{xx}(0)$= longitudinal conductance at zero magnetic field (H), $G_{xx} = \frac{R_{xx}}{\left[R_{xy}^2 + R_{xx}^2\right]}$, can be calculated from the experiment; $C_2 = \lim_{H \to 0} \frac{G_{xy}(H)}{eH}$, which is the linear slope of G_{xy} in near zero H.

Figure 3(d) shows the experimentally measured R_{xx} with H from 10 to 200 K, showing negative magnetoresistance (NMR). This NMR is significant at CTs which decreases with the increase in temperature and vanishes after $T \geq 200$ K. The behavior of R_{xx} for $T \geq 200$ K is shown in the Supporting material (Figure S8). The NMR for $T \leq 180$ K could be a signature of the presence of IB conduction, which is also observed in other doped crystalline semiconductors[4-7]. Figure 3(d) also shows experimentally measured G_{xy} (H) at different temperatures along with the fitting of the two-band model (represented by the yellow solid line) using Equation (4), with fixed experimentally measured values of C_1 and C_2 and initial values of μ_1 and μ_2. Next, using C_1, C_2, μ_1, and μ_2, the values of n_1 and n_2 were determined using Equations (5) and (6). The derived values of n_1, n_2, μ_1, and μ_2 with temperatures are shown in Supporting material (Figure S9). Using these

values, the values of ρ and R_H were calculated with Equations (1) and (2), which match exactly with measured values as shown in red rhombus in Figure 3(b) and (c).

Both the two-band model and measured value show a reduction in R_H for T < 140 K, which suggests the domination of IB[8]. Hence, both NMR (from R_{xx}) and two-band model analysis confirm the presence of IB conduction in β-Ga_2O_3 films and thereby in FinFETs at temperature T ≤ 180 K with IB domination at T < 140 K. The theoretical framework of CB to IB conduction transition is discussed in the Supporting material (Section XII). Figure 3(e) summarizes three conduction regions in the β-Ga_2O_3 films based on above analysis: (I) $T \geq 200$ K: "CB conduction"; (II) 140 ≤ T ≤ 180 K: "Onset of IB"; (III) T ≤ 140 K: "IB conduction dominate". Further, the dominance of IB at T < 140 K in the films correlates with the observed increase in the SS at T < 150 K in the β-Ga_2O_3 FinFETs. The localized states associated with IBs introduce an additional capacitance component $C_{localized}$, at CTs, which becomes a primary factor contributing to increase in SS, particularly when the electron conduction is governed by IBs. Further analysis is provided in the Supporting material (Section XIII).

Further, the mechanism of electron conduction in IB can be understood by fitting the $\rho(T)$ with 3D Mott's Equation as following Equation (7)[Error! Bookmark not defined.].

$$\rho = \rho_{o,mott}\, exp\, (\left(\frac{T}{T_o}\right)^{-\frac{1}{4}})\,(7)$$

Mott's equation fitted well (red line) with experimentally measured ρ values as shown in Figure 3(g) which confirms the 3D VRH is the mechanism for electron conduction in IB, similar to those observed in other β-Ga_2O_3 doped substrates[Error! Bookmark not defined.]. Here, the T_o is the characteristic temperature that leads to the localization length of ~10 nm[9]. Further details are discussed in Supporting material (Section XIV).

Section X. Longitudinal resistance (R_{xx}) for T > 200 K

The variation of R_{xx} in the presence of magnetic field (H) ranging from $-8 \leq H \leq 8$ *Tesla* for temperature (*T*) above 200 Kelvin (K) is shown in Figure S8. It exhibits negligible magnetoresistance compared to those of $T < 200$ K, indicating the dominance of the conduction band in electron conduction for $T \geq 200$ K.

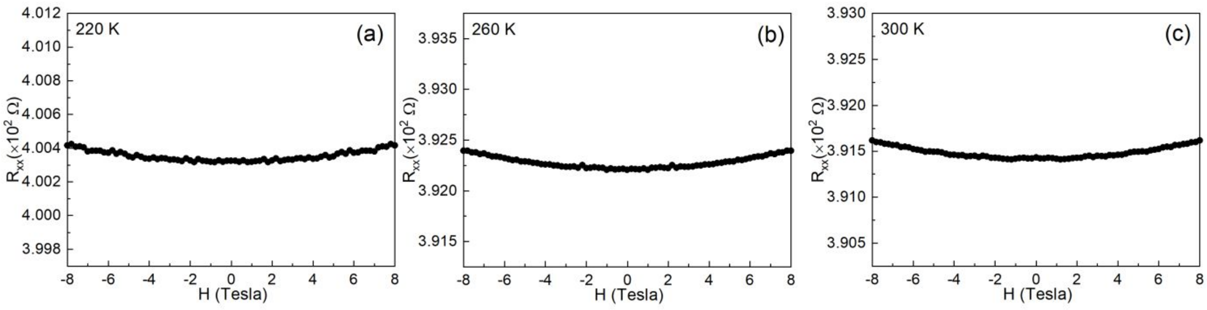

Figure S8. R_{xx} of β-Ga_2O_3 at (a) 220 K, (b) 260 K, and (c) 300 K.

Section XI. Two-band model

The experimental measurement of Hall conductance (G_{xy}) was fitted using a two-band model to obtain the values of μ_1, μ_2, as well as n_1, n_2. The variations of n_1, n_2, μ_1, and μ_2 are illustrated in Figure S9. Here, (n_1, μ_1) and (n_2, μ_2) represent the sheet carrier concentration and bulk mobility in the conduction band (CB) and the intermediate band (IB), respectively.

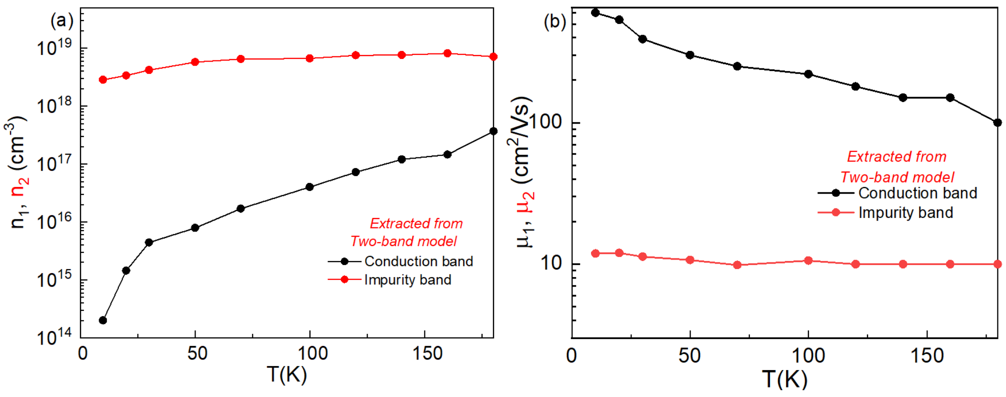

Figure S9. The value of (a) n_1, n_2 and (b) μ_1, μ_2 at different temperatures; extracted from the fitting of G_{xy} using the two-band model (from Equation (4), (5), (6), main manuscript).

Section XII. Theoretical Framework for CB to IB Conduction Transition

To quantify the CB to IB transition at cryogenic temperatures (CTs), we estimate the activation energy (E_A) associated with electron excitation from the impurity band to the conduction band. This is achieved by fitting the temperature dependence of the carrier concentration (n) using an Arrhenius Equation. As shown in Figure S10, the fitting is performed in the high-temperature range ($T > 180$ K), where conduction is primarily via the CB, consistent with our two-band model discussed in the manuscript.

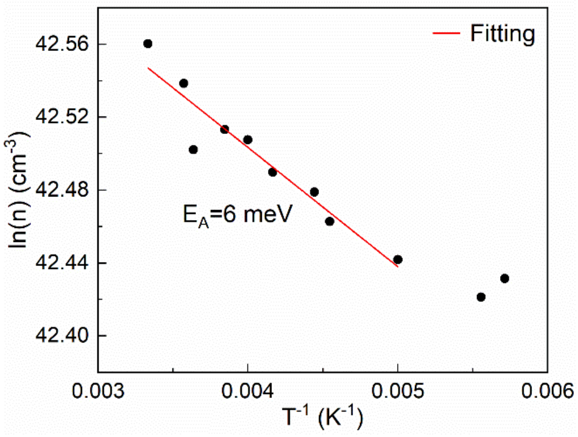

Figure S10. Arrhenius plot of electron concentration (n) versus inverse temperature ($1/T$), showing a linear relationship in the *ln(n)* vs $1/T$ plot, indicating activation energy.

From the linear fit of ln(n) versus $1/T$, we extract an E_A of ~6 meV. This energy corresponds to the separation between the conduction band minimum and the impurity band maximum (i.e., $E_C - E_{IB}$). This low value of E_A is consistent with prior studies on highly doped β-Ga_2O_3 thin films[10].

Due to the small E_A, impurity band conduction begins to contribute significantly at intermediate temperatures (~180 K) and becomes dominant at T < 140 K. The transition in the dominant

conduction mechanism from CB to IB is schematically illustrated using the energy band diagrams in Figure S11.

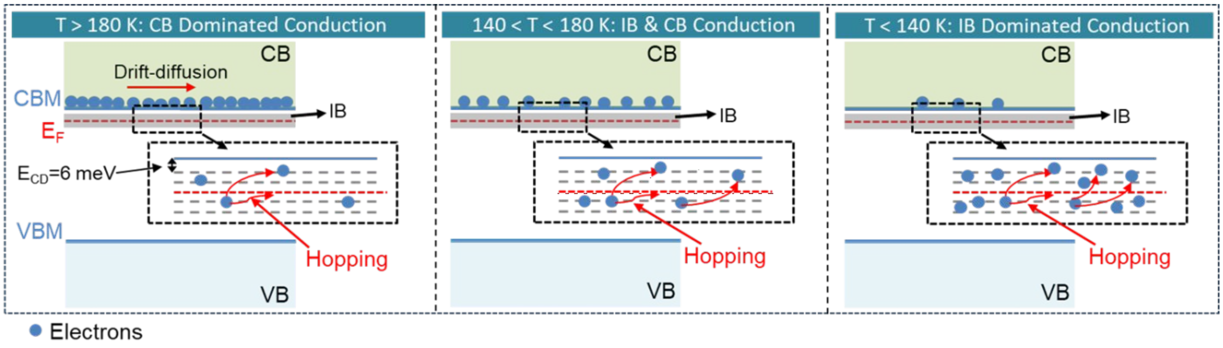

Figure S11. Illustration of electron conduction mechanisms across three temperature regions ($T > 180$ K, 140 K $< T <$ 180 K, and $T < 140$ K) based on the two-band model. Conduction is dominated by the conduction band for $T > 180$ K and by the impurity band for $T < 140$ K.

Conduction Mechanism with Temperature:

For $T > 180$ K: Most electrons are thermally excited into the conduction band, and transport is dominated by CB conduction.

For 140 K $< T <$ 180 K: Both CB and IB contribute, and a transition regime is observed.

For $T < 140$ K: The thermal energy is insufficient to excite electrons into the CB. Electrons instead transport via impurity band conduction, likely governed by variable range hopping (VRH) among localized states within the IB.

Section XIII. Analysis of Abnormal Behavior of Subthreshold Swing

The subthreshold swing (*SS*) behavior is influenced by both the presence of impurity bands (IBs) and interface traps. As discussed in the manuscript, the IB consists of localized states contributed by Si impurities/dopants in β-Ga_2O_3, which are located near the conduction band minimum. The

density of these localized states ($D_{localized}$) can be considered analogous to interface traps, as electrons are trapped in these states near the MOS interface, depending on the position of the Fermi level, as illustrated in the band diagram (Figure 4, main manuscript). Consequently, the charge distribution associated with these localized states can induce a temperature dependence of the subthreshold swing (*SS*), similar to that caused by conventional interface traps.

Therefore, the $D_{localized}$ can induce an additional capacitance $C_{localized}$ in the SS Equation, as shown in (S5)[11,12].

$$SS = \frac{K_B T}{q} ln(10) \times (1 + \frac{C_{it}+C_{localized}}{C_{ox}}) \qquad \text{(S5)}$$

where C_{it}, $C_{localized}$, and C_{ox} are the capacitance due to oxide-semiconductor interface traps, localized states, and gate oxide, respectively.

As confirmed by the two-band model analysis discussed in the manuscript, electron conduction occurs primarily through the conduction band for $T > 150$ K. In this regime, the contribution to the *SS* from localized states (i.e., $C_{localized}$) is negligible, resulting in *SS* being predominantly influenced by the interface trap C_{it}.

In contrast, at cryogenic temperatures ($T < 150$ K), C_{it} becomes negligible due to the freeze-out of interface traps D_{it}, as also evidenced by the observation of negligible voltage hysteresis. However, $C_{localized}$ becomes dominant due to the increasing influence of impurity band (IB) conduction, leading to an increase in *SS*, as described by Equation (S6)[11]. This equation is applicable only when electron transport is dominated by IB conduction, which is valid in the regime where $T << W_t/K_B$.

$$SS = \frac{W_t}{q} ln(10) \times \left(1 + \frac{C_{localized}}{C_{ox}}\right) \quad at\ T << W_t/K_B \qquad \text{(S6)}$$

$$= \frac{W_t}{q} ln(10) \times (1 + \frac{qD_{localized}}{C_{ox}})$$

Here, W_t represents the energy width of the tail states of the impurity band (IB), defined as the width of an exponentially decaying function that describes the energy distribution of the entire tail density of states (DOS_{tail})[11,12]. Since IB conduction becomes dominant at $T < 150$ K, so we use this temperature to estimate $W_t = \frac{K_B T}{e} = 13$ meV.

To evaluate the contribution of $D_{localized}$ to the *SS*, we select $T = 2$ K for the SS analysis at CTs, as it serves two key purposes: (i) the condition $T << W_t/K_B$ is satisfied, and (ii) the contribution of D_{it} to SS can be neglected due to the complete freeze-out of interface trap charges.

The value of $D_{localized}$ can be calculated by multiplication of doping concentration (n) of ~5×10^{18} cm^{-3} and localization length (ξ) of ~10 nm at 2 K[13]. The ξ is calculated using Mott's variable range hopping as discussed in Section XII of the supplementary materials. By substituting the value of $D_{localized}$, C_{ox}, and W_t, the value of SS at 2 K is:

$$SS\ (2\ K) = \frac{13\ meV}{e} \times 2.30 \times (1 + \frac{1.6 \times 10^{-19} \times 5 \times 10^{18} \times 10 \times 10^{-7}}{1.88 \times 10^{-7}})$$

The calculated *SS* value at 2 K is ~156 mV/dec, which is near to the measured SS value of 152 mV/dec at 2 K.

In summary, the SS depends on both $D_{localized}$ of IBs and D_{it} of oxide-semiconductor interface, which dominate at cryogenic and high temperatures, respectively. The dominance of $D_{localized}$ at CTs for $T < 150$ K contributes to increased *SS*, confirming consistency with the experiment results.

Section XIV. 3D Mott Variable range hopping (VRH) and localization effect

The resistivity (ρ) curve was fitted with 3D Mott VRH (Equation 7, main manuscript) which provides the Mott's characteristics temperature, T_o. This leads to the calculation of localization length (ξ), using Equation (S8)[13]. From fitting, the value of T_o is ~ 41 K.

$$\xi = \left(\frac{\beta}{k_B g(E_F) T_O}\right)^{1/3} \quad \text{(S8)}$$

where β=18, a numeric parameter, $g(E_F)$ is the density of states at fermi energy, and ξ is the localization length.

The value of $g(E_F)$ is 8.3×10^{19} eV^{-1} cm^{-3}, calculated using electron concentration and Rydeberg energy. Considering the value of $g(E_F)$ and T_o, the value of ξ is ~ 10 nm.

The localization length (ξ) and the average distance between two Si atoms in the β-Ga_2O_3 channel are comparable. This observation confirms that electron hopping between localized states induces impurity band conduction, which serves as the primary conduction mechanism at cryogenic temperatures (CTs). As temperature decreases, this localization effect becomes increasingly dominant. Consequently, the overlap of electron wavefunctions becomes less certain, leading to the most probable transport mechanism at CTs being variable-range hopping (VRH).

Section XV. Correlation of V_{TH} shift with Freeze-out of Interface Trap

Interface traps or states at the oxide–semiconductor interface play a crucial role in determining the electrical characteristics of MOSFETs, such as the threshold voltage (V_{TH}), subthreshold swing (*SS*), and hysteresis voltage (ΔV_{HY}). In the following discussion, we will refer to these interface traps/states as D_{it}. At the Al_2O_3/Ga_2O_3 interface, D_{it} are typically positively charged when

unoccupied, and become neutral upon capturing electrons[14]. This behavior is supported by the observed clockwise hysteresis in the transfer characteristics.

When D_{it} are occupied by electrons, they reduce the free electron concentration in the active channel, contributing to a positive shift in V_{TH}. The occupancy of these traps is temperature-dependent, governed by their activation energy. Measuring ΔV_{HY} across different temperatures—while maintaining a constant voltage sweep range—can serve as a reliable method to assess D_{it} occupancy.

As shown in Figure 2(c), ΔV_{HY} decreases monotonically with decreasing temperature. This trend indicates that electron trapping and de-trapping in D_{it} diminish at lower temperatures, confirming that more traps become permanently occupied by electrons. Furthermore, these trapped electrons cannot be released by gate voltage sweeps at CTs, indicating that the traps have effectively frozen out. This freeze-out reduces the available electron concentration in the active channel, thus requiring a higher gate voltage to turn on the device, resulting in a positive V_{TH} shift.

This behavior can also be explained in terms of the charge states of $D_{it,}$ which are referred by interface trap charges (N_{it}). When traps are empty, they are positively charged N_{it} and contribute an additional electric field that aids in forming the conduction channel, leading to an earlier device turn-on. However, when traps are neutral (i.e., filled with electrons), this additional field is absent, requiring a higher gate voltage to turn on the MOSFET, resulting a positive V_{TH} shift.

<u>Theoretical Explanation via Energy Band Diagrams:</u>

The observation of V_{TH} shift with occupation and freeze-out of interface traps can be understood by the depletion and accumulation band diagram at 300 and 2 K, as shown in Figure 4 (main

manuscript). The interface states, $D_{it,}$ are the same irrespective of temperatures, but occupation of these D_{it}, referred to as $N_{it,}$ becomes different with temperatures.

The difference in the occupation of $D_{it,}$ shown as $N_{it,}$ can be observed in the depletion region of the band diagram, as in the accumulation region, all D_{it} are filled with electrons. At 300 K, most D_{it} states remain unoccupied, contributing to a positive N_{it} and significant hysteresis voltage (ΔV_{HY}). However, as the temperature decreases, these traps capture electrons and are unable to release them during the same negative voltage sweep range, resulting in the freeze-out of D_{it} and reduction in N_{it}, as illustrated in Figure R7. This freeze-out leads to a positive shift in the threshold voltage (V_{TH}).

Section XVI. Quantitative Analysis: Positive shift in threshold voltage (V_{TH}) at CTs

The D_{it} at 300 K is ~1.59×10^{12} cm^{-2} eV^{-1} extracted using the subthreshold swing (*SS*) equation at 300 K, as shown in Equation (8) of the main manuscript. Based on the energy band diagram discussed above, it is evident that the occupation of these interface traps by electrons at cryogenic temperatures (CTs) contributes to the positive V_{TH} shift.

However, directly calculating D_{it} at CTs is not straightforward, as localized states in the impurity band dominate at low temperatures. These states introduce an additional capacitance in the SS equation, resulting in increased SS (see Supplementary Material, Section XI). Therefore, an alternative approach using voltage hysteresis measurements provides a useful estimation of occupation of D_{it} i.e. N_{it} at 2 K.

During gate voltage sweeps, interface traps can capture or release electrons, and this dynamic behavior is reflected in the hysteresis voltage (ΔV_{HY}). A significantly reduced ΔV_{HY} at 2 K suggests that fewer interface traps are available for charge exchange, indicating that most traps

are already filled (i.e., neutral). Hence, the positively charged interface traps, N_{it} at 2 K, can be estimated by correlating it with ΔV_{HY} using the following proportionality:

$$N_{it} \propto V_{HY} \quad \text{(S9)}$$

$$\frac{(N_{it})_1}{(N_{it})_2} = \frac{(V_{HY})_1}{(V_{HY})_2}$$

Where $(N_{it})_1$, $(N_{it})_2$ is the occupation of D_{it} and $(V_{HY})_1$ and $(V_{HY})_2$ is the hysteresis voltage at 300 and 2 K. Here, $(N_{it})_1$ can be considered equal to D_{it} at 300 K, as SS Equation only estimates the charged D_{it} which contributes to V_{TH}.

So,

$$(N_{it})_2 = \frac{(V_{HY})_2}{(V_{HY})_1} \times (D_{it})_1$$

$$= \frac{0.002}{0.22} \times 1.59 \times 10^{12} \sim 10^{10}\ \text{cm}^{-2}\text{eV}^{-1}$$

So, the change in the N_{it} (ΔN_{it}) from 300 to 2 K is ~1.5×10^{12} cm^{-2} eV^{-1}.

The V_{TH} is defined as the applied gate voltage required to achieve the electron accumulation in the semiconductor channel. This voltage drop occurs across both gate oxide and semiconductors. By considering the positive charges at the Al_2O_3/Ga_2O_3 interface[14] and applying the principle of charge conservation across gate-to-semiconductor, the electron accumulation at V_{TH} can be expressed through Equation (S10)[15].

$$Q_{mT} + Q_{ss} = Q_n \qquad \text{(S10)}$$

Where Q_{mT} is the charge on metal at the threshold, Q_{ss} is the positive charge at Al_2O_3/Ga_2O_3 interface, and Q_n is the electron accumulated in the semiconductor at the threshold.

On the other hand, the V_{TH} of the transistor can be written as Equation (S11).

$$V_{TH} = \frac{Q_{mT}}{C_{ox}} + \phi_{ms} + \phi_s \qquad \text{(S11)}$$

where ϕ_{ms} is metal-semiconductor work function and ϕ_s is surface potential of semiconductors.

Using Equation (S10), (S11) can be re-written as

$$V_{TH} = \frac{Q_n - Q_{ss}}{C_{ox}} + \phi_{ms} + \phi_s \qquad \text{(S12)}$$

The Q_n, C_{ox}, ϕ_{ms}, and ϕ_s is constant with temperature as quantities like electron concentration (from Hall-effect measurement), dielectric constant, and metal and semiconductor work-function are temperature independent.

Therefore,

$$\Delta V_{TH} = \left|\frac{\Delta Q_{ss}}{C_{ox}}\right| \qquad \text{(S13)}$$

As estimated from the *SS* equation: $\Delta Q_{ss} = Q_{it} = (e \times \Delta N_{it}) = 2.4\times10^{-7}$ C/cm^2 between 300 and 2 K. This will lead to ΔV_{TH} ~ 1.1 V using Equation (S13), consistent with the experimental results.

Section XVII. Additional Experiments: Validating for Interface Trap Frezze-out

UV-assisted β-Ga_2O_3 FinFET Measurement at CTs

Ultraviolet (UV) light exposure has been widely employed to modulate the occupation of interface states in ultra-wide bandgap (UWBG) β-Ga_2O_3 MOSFETs[16,17]. In particular, the Al_2O_3/Ga_2O_3 interface states detrap the electrons and become a positively charged (i.e., empty) state under UV exposure. To investigate the occupation and freeze-out behavior of interface traps at CTs, β-Ga_2O_3 FinFETs were measured FinFETs were measured at 77 K both with and without UV illumination.

Devices were tested inside a low-vacuum chamber with incident UV light (wavelength: 365 nm) introduced through a top glass window.

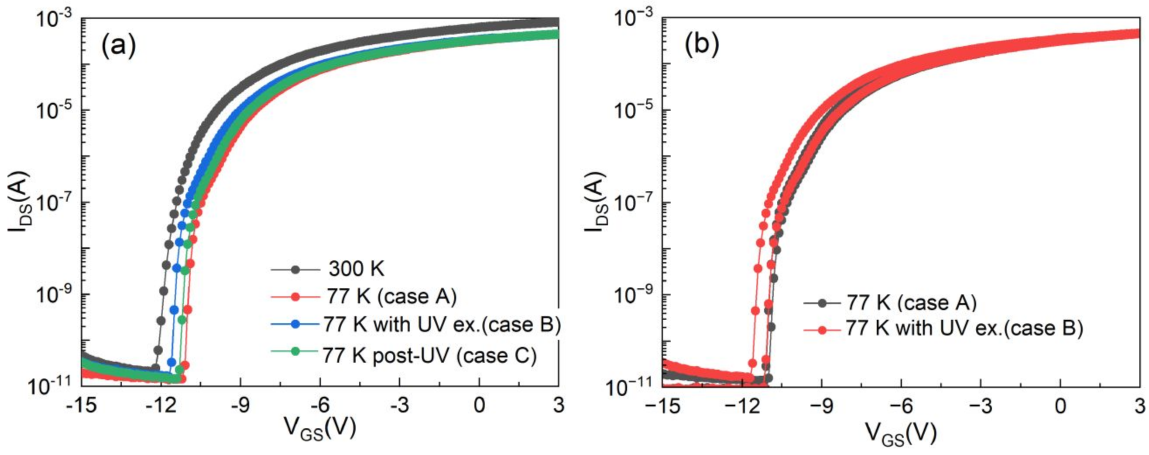

Figure S12. Ultraviolet (UV)-assisted electrical characterization of depletion-mode (*D*-mode) β-Ga_2O_3 FinFETs at 77 K:(a) Transfer characteristics (I_{DS}–V_{GS}) measured before UV exposure, during UV exposure, and after UV exposure. (b) Hysteresis behavior in the transfer curves, comparing measurements taken before and during UV exposure.

Figure S12(a) presents the transfer characteristics of D-mode β-Ga_2O_3 FinFETs at 300 K and 77 K under the following three conditions:

(i) Without UV exposure (Case A)

(ii) Under continuous UV illumination (Case B)

(iii) Post UV exposure, with the UV source turned off (Case C)

A positive V_{TH} shift is observed when cooling from 300 K to 77 K, consistent with results discussed in the main manuscript. However, a noticeable negative V_{TH} shift is observed under UV illumination (Case B) compared to the dark condition (Case A). After the UV source is turned off (Case C), the device characteristics revert back to those observed in Case A.

This behavior indicates that, in the absence of UV light (Case A), a portion of interface traps are frozen out and unable to de-trap electrons, leading to a positive V_{TH} shift. Upon UV exposure (Case B), these traps receive sufficient energy to de-trap electrons, becoming active and freeze-out-free. This results in increased electron availability in the channel, thereby causing a negative V_{TH} shift. When UV light is removed (Case C), these traps re-occupy electrons and freeze again, restoring the positive V_{TH} shift.

This modulation of interface trap activity is further supported by hysteresis measurements shown in Figure S12(b). A higher ΔV_{HY} is observed under UV exposure (Case B) compared to the dark condition (Case A), confirming that more interface states are accessible for charge trapping during accumulation.

This study validates the following points:

(i) The modulation of electron occupancy in Al_2O_3/Ga_2O_3 interface states plays a significant role in determining both the V_{TH} shift and ΔV_{HY}.

(ii) The behavior of ΔV_{HY} and V_{TH} shifts is strongly correlated with the occupation of D_{it} at CTs, supporting the use of ΔV_{HY} as a metric for estimating charged D_{it} i.e. N_{it} (see Equation S9). A higher ΔV_{HY} implies more empty or active interface states, leading to a negative V_{TH} shift, and vice versa.

(iii) The observed characteristics in UV and post-UV measurements confirm that interface traps become frozen at CTs, which is the primary cause of the temperature-dependent trends in V_{TH} and ΔV_{HY}.

Section XVIII. Design of *β*-Ga_2O_3 *n*-channel metal oxide semiconductor (NMOS) inverter integrated circuit (ICs)

To achieve a full output voltage swing from 0 V (logic 0) to V_{dd} (logic 1) with input voltages ranging only between 0 and V_{dd}, the inverter employs an E-mode FinFET with a higher positive V_{TH} as the driver transistor. The E-mode device characterized in the manuscript exhibits V_{TH} values of approximately 0.74 V and 1.87 V at 300 K and 2 K, respectively. Using this device directly in the inverter would require the input voltage to swing below 0 V to fully switch the transistor off, which complicates the biasing scheme and increases circuit complexity[18].

By selecting a driver transistor with a higher positive V_{TH}, the inverter can operate ideally with input voltages strictly between 0 and V_{dd}. This simplifies the biasing conditions by referencing both the input and supply voltages to ground, which improves noise margins, enhances repeatability, and increases the reliability of logic transitions[18].

The *β*-Ga_2O_3 NMOS inverter ICs were fabricated using the monolithic integration of depletion (*D*-) and enhancement (*E*-) mode FinFETs, used as load and driver transistors, respectively, shown in Figure S13. The driver-to-load resistance ratio (α) is defined by Equation (S14).

$$\alpha = \frac{(\frac{W_{CH}}{L_{CH}})_E}{(\frac{W_{CH}}{L_{CH}})_D} \quad \text{(S14)}$$

where W_{CH} and L_{CH} are the channel width and length of the FinFETs, respectively.

For realizing the D- and E-mode FinFETs, the fin width (W_{fin}) of 75 nm and 150 nm were used, respectively. Additionally, the E-mode FinFETs have ~100 fins whereas D-mode FinFETs have only ~2 fins to make sure the value of α~50 (keeping the L_{CH} constant).

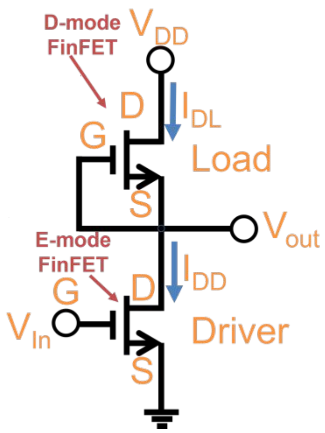

Figure S13. Schematic diagram depletion load n-channel metal oxide semiconductor (NMOS) inverter ICs using D- and E-mode FinFETs.

Benchmarking

Figure S14(a) and (b) show the benchmarking of I_{ON}/I_{OFF} and SS of our *D*- and *E*-mode Ga_2O_3 FinFET with other Ga_2O_3 FinFETs at 300 and 2 K. This work extends the functionality of Ga_2O_3 electronics at CTs, which makes a noteworthy contribution to Ga_2O_3 field, positioning it as a robust contender and an ideal choice for cryogenic and thereby, extreme temperature/environment electronics.

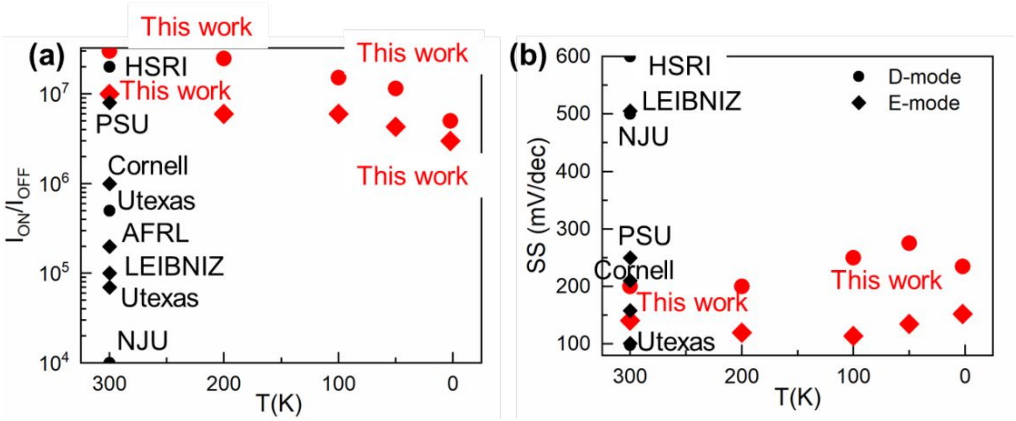

Figure S14. D- and E-mode Ga_2O_3 FinFET parameters at cryogenic temperaures for (a) I_{ON}/I_{OFF} (b) SS, benchmarked with other β-Ga_2O_3 literature.

References

[1] H.-C. Huang, M. Kim, X. Zhan, K. Chabak, J. D. Kim, A. Kvit, D. Liu, Z. Ma, J.-M. Zuo, and X. Li, "High Aspect Ratio β-Ga2O3 Fin Arrays with Low-Interface Charge Density by Inverse Metal-Assisted Chemical Etching," *ACS Nano*,13, 8784 (2019).

[2] V. Khandelwal, S. Yuvaraja, G. I. M. García, C. Wang, Y. Lu, F. AlQatari, X. Li, "Monolithic ***β***-Ga_2O_3 NMOS IC based on heteroepitaxial E-mode MOSFETs," *Applied Physics Letters,* 122, 14, 143502 (2023).

[3] N. Bansal, Y. S. Kim, M. Brahlek, E. Edrey, S. Oh, "Thickness-Independent Transport Channels in Topological Insulator Bi2Se3 Thin Films," *Physical Review Letters*, 109, 11, 116804 (2012).

[4] J. Mukherjee, B. R. K. Nanda, M. S. R. Rao, "Metal–insulator transition in epitaxial Ga-doped ZnO films via controlled thickness" *Journal of Physics: Condensed Matter*, 10, 33, 0953 (2021).

[5] Z. Dziuba, T. Przesławski, K. Dybko, M. Górska, J. Marczewski, K. Regiński, "Negative magnetoresistance and impurity band conduction in an $In_{0.53}Ga_{0.47}As$/InP heterostructure," *Journal of Applied. Physics,* 85, 9, 6619 (1999).

[6] X. Wang, Q. Shao, A. Zhuravlyova, "Giant negative magnetoresistance in Manganese-substituted Zinc Oxide," *Scientific Reports,* 5, 9221 (2015).

[7] J. F. Woods, C. Y. Chen, "Negative Magnetoresistance in Impurity Conduction," *Physical Review*, 135, 5A, A1462 (1964).

[8] H. Fritzsche, M. Cuevas, "Impurity Conduction in Transmutation-Doped p-Type Germanium" *Physical Review*, 119, 4, 1238 (1960).

[9] F. Blanda, "Quantum properties and magnetotransport in β-Ga2O3 thin films" *Master Thesis* (2023).

[10] Z. Kabilova, C. Kurdak, R. L Peterson, "Observation of impurity band conduction and variable range hopping in heavily doped (010) β-Ga2O3" *Semiconductor Science and Technology*, 3, 34, 0268 (2019).

[11] M.-S. Kang, K. Toprasertpong, H. Oka, T. Mori, M. Takenaka, S. Takagi; Characterization and quantitative understanding of subthreshold swing of Si metal–oxide–semiconductor field effect transistors at cryogenic temperatures, J. Appl. Phys., 136, 195702 (2024).

[12] A. Beckers, F. Jazaeri and C. Enz, "Inflection Phenomenon in Cryogenic MOSFET Behavior," in IEEE Transactions on Electron Devices, 67, 3, 1357, (2020).

[13] F. Blanda, "Quantum properties and magnetotransport in β-Ga2O3 thin films" *Master Thesis* (2023).

[14] D. Biswas, C. Joishi, J. Biswas, K. Thakar, S. Rajan, S. Lodha, “Enhanced *n*-type β-Ga_2O_3 (-201) gate stack performance using Al_2O_3/SiO_2 bi-layer dielectric”, *Applied Physics Letters,* 27, 114, 212106 (2019).

[15] D. A. Neamen, and D. Biswas, “Semiconductor physics and devices” *McGraw-Hill higher education*, United States (2011).

[16] H. Zhou, S. Alghmadi, M. Si, G. Qiu, and P. D. Ye. "Al2O3/$\beta $-Ga2O3 (-201) Interface Improvement Through Piranha Pretreatment and Postdeposition Annealing." IEEE Electron Device Letters 37, 11, 1411(2016).

[17] Z. A. Jian, S. Mohanty, and E. Ahmadi. "Deep UV-assisted capacitance–voltage characterization of post-deposition annealed Al2O3/β-Ga2O3 (001) MOSCAPs." Applied Physics Letters 116, 24 (2020).

[18] Pingqiang Zhou, "EE213 Digital Integrated Circuits II Lecture 5: CMOS Inverter," ShanghaiTech University, School of Information Science and Technology, Fall 2018. Available: https://faculty.sist.shanghaitech.edu.cn/faculty/zhoupq/Teaching/Fall18/Lectures/EE213-lec5.pdf**
